# The Evolution of Cytogenetic Traits in *Cuscuta* (Convolvulaceae), the Genus With the Most Diverse Chromosomes in Angiosperms

**DOI:** 10.3389/fpls.2022.842260

**Published:** 2022-04-01

**Authors:** Amalia Ibiapino, Miguel A. García, Bruno Amorim, Mariana Baez, Mihai Costea, Saša Stefanović, Andrea Pedrosa-Harand

**Affiliations:** ^1^Laboratory of Plant Cytogenetics and Evolution, Department of Botany, Federal University of Pernambuco, Recife, Brazil; ^2^Real Jardín Botánico-CSIC, Madrid, Spain; ^3^Postgraduate Program of Biotechnology and Natural Resources of the Amazonia (PPGMBT), State University of Amazonas, Manaus, Brazil; ^4^Plant Breeding Department, University of Bonn, Bonn, Germany; ^5^Department of Biology, University of Wilfrid Laurier, Waterloo, ON, Canada; ^6^Department of Biology, University of Toronto Mississauga, Mississauga, ON, Canada

**Keywords:** character evolution, ancestral chromosome number, genome size, ribosomal DNA, heterochromatin, karyotype evolution

## Abstract

Karyotypes are characterized by traits such as chromosome number, which can change through whole-genome duplication and dysploidy. In the parasitic plant genus *Cuscuta* (Convolvulaceae), chromosome numbers vary more than 18-fold. In addition, species of this group show the highest diversity in terms of genome size among angiosperms, as well as a wide variation in the number and distribution of 5S and 35S ribosomal DNA (rDNA) sites. To understand its karyotypic evolution, ancestral character state reconstructions were performed for chromosome number, genome size, and position of 5S and 35S rDNA sites. Previous cytogenetic data were reviewed and complemented with original chromosome counts, genome size estimates, and rDNA distribution assessed *via* fluorescence *in situ* hybridization (FISH), for two, seven, and 10 species, respectively. Starting from an ancestral chromosome number of *x* = 15, duplications were inferred as the prevalent evolutionary process. However, in holocentric clade (subgenus *Cuscuta*), dysploidy was identified as the main evolutionary mechanism, typical of holocentric karyotypes. The ancestral genome size of *Cuscuta* was inferred as approximately 1C = 12 Gbp, with an average genome size of 1C = 2.8 Gbp. This indicates an expansion of the genome size relative to other Convolvulaceae, which may be linked to the parasitic lifestyle of *Cuscuta*. Finally, the position of rDNA sites varied mostly in species with multiple sites in the same karyotype. This feature may be related to the amplification of rDNA sites in association to other repeats present in the heterochromatin. The data suggest that different mechanisms acted in different subgenera, generating the exceptional diversity of karyotypes in *Cuscuta*.

## Introduction

Eukaryotes vary in their chromosome constitution and are often characterized by their karyotypes, including both chromosome number and morphology. Among flowering plants, chromosome number has a wide range of variation from 2*n* = 4 to 2*n* = 640 (Uhl, 1978; Roberto, 2005). The distribution of chromosome numbers in any given monophyletic group allows the identification of one or more chromosome numbers that are considered the ancestral haploid number or basic number of each clade, referred to as *x* (Guerra, 2008; Mayrose and Lysak, 2021).

From an evolutionary perspective, changes in chromosome number can occur through several mechanisms among which whole-genome duplications within a lineage or autopolyploidy is of special importance (Heslop-Harrison and Schwarzacher, 2011; Alix et al., 2017; Mayrose and Lysak, 2021). Polyploidy can also result from a hybridization event involving two different lineages, a process through which allopolyploids are established (Qiu et al., 2020). Another important source of changes involves ascending and descending dysploidy, that is, a stepwise gain and loss of chromosomes due to structural rearrangements. Descending dysploidy results from incorrect double-stand break repair in two or more chromosomes resulting in chromosome fusion by translocation. This fused chromosome can be inherited by the offspring. In monocentric chromosomes, fusion is usually followed by the elimination or inactivation of one of the centromeres (Guerra, 2008; Schubert and Lysak, 2011; Carta et al., 2020; Mayrose and Lysak, 2021). Centric fission is considered the most common type of ascending dysploidy. The break within a centromere or the wrong centromeric division gives rise to two chromosomes that will be inherited if their function is not impaired (Mayrose and Lysak, 2021). While dysploidy increases or decreases the number of chromosomes mostly preserving the genetic content, aneuploidy is the addition or deletion of one or more chromosomes. Aneuploids can originate in a variety of ways, with mis-segregation during meiosis or mitosis being the most common cause (e.g., Mandáková and Lysak, 2018). The establishment of aneuploids is considered to be uncommon because of imbalance in gene dosage, irregular meiosis, and loss of fertility (Mayrose and Lysak, 2021). Molecular phylogenies have contributed not only to estimate the ancestral chromosome number of a particular clade, but also to our understanding of the polarity of chromosome changes.

In addition to the chromosome number, the evolution of different karyotype features, such as chromosomal bands, number and distribution of ribosomal DNA (rDNA) sites, and genome size can be understood in the light of evolution within a clade and can be correlated, among others, to species diversification (Vaio et al., 2013; Costa et al., 2017; García et al., 2017; Sader et al., 2019). These analyses are performed by mapping and comparing cytogenetic data within phylogenetic trees. The integration of phylogenetic and cytogenetic data also enables reconstructing the ancestral states of cytogenetic characters, evaluating different scenarios of trait evolution. Methods based on parsimony or, more commonly, on probabilistic models have allowed to test chromosome evolution hypotheses within a phylogenetic context, determining characters such as ancestral chromosome numbers (Revell, 2012; Glick and Mayrose, 2014; Maddison and Maddison, 2018; Rice and Mayrose, 2021). Tools such as ChromEvol can estimate the ancestral chromosome number along each branch of a phylogeny while also inferring events like polyploidy and dysploidy (Glick and Mayrose, 2014). Other analytical tools, for example, the R package phytools, allow the reconstruction of ancestral genome size (Revell, 2012), or Mesquite, which was used to reconstruct ancestral states of any characters, including the number and position of heterochromatic bands, as well as 5S and 35S rDNA sites (Revell, 2012; Glick and Mayrose, 2014; Maddison and Maddison, 2018). These approaches are particularly relevant when dealing with extensive samplings or highly variable groups. More recently, Yoshida and Kitano (2021) have proposed a probabilistic method of karyotype evolution incorporating both chromosome and arm numbers, whereby they allowed for a consideration of chromosome morphology as well.

*Cuscuta* L. (dodders; Convolvulaceae) is a cytogenetically highly diverse genus, with chromosome numbers ranging from 2*n* = 8 to 2*n* = 150 (Pazy and Plitmann, 1995; García and Castroviejo, 2003; García et al., 2019). The basic numbers for the genus were proposed to be *x* = 15 and *x* = 7 (Fogelberg, 1938; García and Castroviejo, 2003). Most species are diploids with 2*n* = 30, but also allopolyploid and autopolyploid species have been documented (García et al., 2014, 2018). Furthermore, among the approximately 200 species of *Cuscuta* (Costea et al., 2015a) genome size varies more than 128-fold, from 1C = 0.27 Gbp in *C. australis* R.Br. to 1C = 34.73 Gbp in *C. reflexa* Roxb. (Sun et al., 2018; Neumann et al., 2020), the highest variation documented for a single genus in angiosperms. This genus is divided into four subgenera, each one with particular cytogenetic features, suggesting strong phylogenetic signals for cytogenetic characters in the group (García et al., 2019; Ibiapino et al., 2022). Subgenus *Cuscuta* is characterized by the presence of holocentric chromosomes; subgen. *Grammica* (Lour.) Yunck. shows the largest variation in chromosome size and number as well as genome size, with confirmed cases of auto- and allopolyploidy; subgen. *Monogynella* (Des Moul.) Peter, Engl. & Prantl includes species with the largest genomes and chromosomes (Fogelberg, 1938; Pazy and Plitmann, 1994; García and Castroviejo, 2003; Guerra and García, 2004; McNeal et al., 2007; García et al., 2019; Ibiapino et al., 2019; Neumann et al., 2020); and finally subgen. *Pachystigma* (Engelm.) Baker & C. H. Wright comprises species with conspicuously bimodal karyotypes. Intraspecific chromosome number variation has been also reported. In species such as *Cuscuta epithymum* (L.) L. and *Cuscuta planiflora* Ten., chromosome number can differ among populations. This variation is even more intriguing in *C. epithymum*, which has holocentric chromosomes and shows 2*n* = 14, 16, 28, 30, 32, and 34 in different populations (García and Castroviejo, 2003).

Given the currently available evidence, *Cuscuta* is the genus with the broadest chromosome diversity of all angiosperms. No other genus has both holocentric and monocentric chromosomes as well as such a diversity in chromosome size and numbers together with up to a 128-fold difference in genome size. Furthermore, this enormous variation is found at a very low (species) phylogenetic level, which makes this lineage a very tractable system to study genome evolution. Only the carnivorous clade of Caryophyllales shows similar karyotypic diversity but lower differences in genome size: holocentric chromosomes in Droseraceae (1C = 0.24–5.46 Gbp), small monocentric chromosomes in Nepenthaceae (1C = 0.67–1.36 Gbp), and big monocentric chromosomes in Drosophyllaceae (1C = 10.42 Gbp; Veleba et al., 2017, 2020). *Cuscuta* is remarkable because it shows more variation in chromosome and genome size than the five families of carnivorous Caryophyllales even though the clade age of the latter is estimated in the late Cretaceous, c. 84 Mya (Biswal et al., 2018), whereas the *Ipomoea*-*Cuscuta* lineages split c. 33 Mya (Sun et al., 2018) and the crown age of *Cuscuta* are estimated at 23.0–20.5 Mya (Neumann et al., 2020).

Other parameters, such as the number and position of heterochromatic bands and 5S and 35S rDNA sites, have been comparatively less studied. Nevertheless, the few species investigated still revealed an enormous variation. *Cuscuta denticulata* Engelm. showed one pair of CMA^+^/DAPI^+^ bands, one pair of 5S rDNA, and one pair of 35S, while *C. monogyna* Vahl presented at least 90 CMA^+^ bands, 80 DAPI^+^ bands, 36 5S rDNA sites, and 30 35S rDNA sites (Ibiapino et al., 2019, 2020).

Taken together, this striking karyotypic variation combined with a well-resolved phylogeny (García et al., 2014) makes *Cuscuta* an excellent model for studying karyotypic evolution events in flowering plants. Therefore, the aim of this work was to reconstruct the ancestral states for characters such as chromosome number, genome size, and the position of ribosomal DNA sites in the genus *Cuscuta*. To this end, we reviewed all available data and expanded the banding and rDNA distribution data for 10 previously unstudied species from different clades, six new genome size estimates, and two new chromosome counts, to understand how karyotype evolution occurred and to infer the main events involved in these changes within each subgenus and among subgenera. We also provide a comparative overview of the evolution of genome size and its relationship to the parasitic lifestyle of the genus in a phylogenetic framework.

## Materials and Methods

### Sequence Sampling and Phylogenetic Analysis

For the phylogenetic reconstruction of ancestral chromosome numbers, we sampled 58 taxa of 57 species of *Cuscuta*, including *C. indecora* Choisy var. *indecora* and *C. indecora* var. *neuropetala* (Engelm.) Hitch. The subgenera *Cuscuta*, *Grammica*, *Monogynella,* and *Pachystigma* were represented by eight, 42, four, and three species, respectively. While the monophyly of *Cuscuta* was never seriously challenged, its outgroup relationships and the phylogenetic position within Convolvulaceae remain unresolved (Stefanović and Olmstead, 2004). For this reason, the interpretation of character evolution was based on the ingroup distribution of character states similar to other character evolution studies conducted recently in the genus (e.g., Ho and Costea, 2018, references therein). We used a total of 226 sequences of nuclear (nrITS and 26S) and plastid markers (*rbc*L and *trn*L-*trn*F) obtained by Stefanović and Costea (2008) and García et al. (2014) deposited in GenBank database (Benson et al., 2012; Supplementary Table 1). In addition, new ITS and *trn*L-*trn*F sequences were obtained for *C. globosa* Ridl., because this hexaploid was not included in previous phylogenetic works. The methods of DNA extraction, amplification, and sequencing were those detailed in Stefanović et al. (2007). Sequences were uploaded to GenBank with accession numbers OL362011 (ITS) and OL362010 (*trn*L-*trn*F). To align the sequences, the plugin MUSCLE was used in the Geneious v. 7.1.9 software (Kearse et al., 2012).

The phylogenetic relationships were reconstructed using Bayesian Inference (BI) analysis. jModelTest v.2.1.6 (Darriba et al., 2012) selected GTR + I + gamma as the best model of DNA substitution for all analyzed regions, except for *trn*L-*trn*F, which had GTR + gamma as best model. We used MrBayes v. 3.2.6. (Ronquist et al., 2012) to perform BI using the concatenated sequences and selected models with two independent runs with four Markov Chain Monte Carlo (MCMC), sampling every 1,000 generations in a total of 15,000,000 generations. Both BI runs were evaluated in Tracer v.1.6 (Rambaut et al., 2014) to verify if the estimated sample sizes (ESS) for each parameter were higher than 200. The consensus tree was generated in MrBayes with a burn-in of 25%. The consensus tree with the posterior probability (PP) was visualized and edited in FigTree v. 1.4.2. (Rambaut, 2014). The jModelTest and BI analysis were performed through the CIPRES Science Gateway (Miller et al., 2010).

### Slide Preparation and FISH

New data on the number and position of rDNA sites for 10 species of *Cuscuta* were obtained for this study (Table 1, voucher information in García et al., 2019). Young shoot tips or flower buds were used for slide preparation according to Ibiapino et al. (2019). Double CMA/DAPI staining was performed as described in Ibiapino et al. (2022). The images were captured with a COHU CCD camera attached to a Leica DMLB fluorescence microscope equipped with Leica QFISH software. After image capture, slides were destained for 30 min in Carnoy and 1 h in absolute ethanol and stored for *in situ* hybridization at −20°C. The destained slides were subjected to fluorescent *in situ* hybridization (FISH) according to the protocol described in Pedrosa et al. (2002). Two rDNA probes were used as: the PCR amplified insert of D2 from *Lotus japonicus* (Regel) K. Larsen (5S rDNA; Pedrosa et al., 2002) and p*Ta*71 from wheat (25-28S, 5.8S, and 18S rDNA; Gerlach and Bedbrook, 1979). Probes were labeled by nick translation with Cy3-dUTP (5S) and digoxigenin 11-dUTP (35S). The 5S was labeled in a reaction with total volume of 12.5 μl containing 1 μg of PCR amplified DNA, 1× Nick Translation buffer (0.5 M Tris HCl pH 7.5; 50 mM MgCl2), dNTP mix (0.016 mM each of dATP, dCTP, and dGTP), 0.08 mM Cy3-dUTP or Alexa-dUTP, 7.5 U of DNA Polymerase I, and 0.006 U of DNase I. The mixture was incubated at 15°C for 1 h or longer if needed, until most fragments were under 500 bp, and reactions were stopped using 0.5 M EDTA. The 35S was labeled with the Nick Translation kit (Invitrogen). The images were obtained as previously described.

**Table 1:** Data of genome size, 5S and 35S ribosomal DNA sites number, and position in species of the genus *Cuscuta.*

Species	1C (Gbp)	5S/35S^*^/^**^	References (Genome size/rDNA)
*Cuscuta americana*	0.68 and 0.69		Neumann et al., 2020, this study
*Cuscuta approximata*		2T + 4I/2T	Guerra and García, 2004
*Cuscuta australis*	0.27, 0.34 and 0.69	2I/2P	Sun et al., 2018, Neumann et al., 2020, this study/This study
*Cuscuta californica*	0.39		Neumann et al., 2020
*Cuscuta campestris*	0.45, 0.55 and 0.58	4I/2I + 2P	This study, Neumann et al., 2020, Vogel et al., 2018/This study
*Cuscuta cephalanthi*	3.68 and 3.83		This study, McNeal et al., 2007
*Cuscuta chilensis*	2.80		McNeal et al., 2007
*Cuscuta compacta*	3.24 and 7.67		This study, McNeal et al., 2007
*Cuscuta denticulata*		2I/2P	Ibiapino et al., 2019
*Cuscuta epilinum*	1.54 and 3.38		McNeal et al., 2007; Neumann et al., 2020
*Cuscuta epithymum*	0.53 (2*n* = 14)	4I/2T	Neumann et al., 2020/This study
*Cuscuta exaltata*	20.51		McNeal et al., 2007
*Cuscuta europaea*	1.05 and 1.17		McNeal et al., 2007; Neumann et al., 2020
*Cuscuta globosa*	1.79	6I/2I + 2P	This study/ This study
*Cuscuta glomerata*	5.16	2I/2P	This study/This study
*Cuscuta gronovii*	3.58		Neumann et al., 2020
*Cuscuta gronovii* (C PA)	6.75		McNeal et al., 2007
*Cuscuta gronovii* (NJ)	3.70		McNeal et al., 2007
*Cuscuta gronovii* (OH)	3.51		McNeal et al., 2007
*Cuscuta gronovii* (SE PA)	2.14		McNeal et al., 2007
*Cuscuta howelliana*		2I/2P	This study
*Cuscuta indecora*	22.68, 24.46 and 32.05	6I + 4I/4P	Ibiapino et al., 2019; McNeal et al., 2007/Ibiapino et al., 2020 Neumann et al., 2020
*Cuscuta japonica*	25.58		Neumann et al., 2020
*Cuscuta lupuliformis*	21.97		McNeal et al., 2007
*Cuscuta monogyna*	32.45 and 33.05	36/30	Ibiapino et al., 2020/Ibiapino et al., 2020; Neumann et al., 2020
*Cuscuta nevadensis*		6I/8I + 2P	Ibiapino et al., 2019
*Cuscuta nitida*		2I/4P	Ibiapino et al., 2022
*Cuscuta obtusiflora*	0.77		McNeal et al., 2007
*Cuscuta partita*	1.83	2I/2P	This study/This study
*Cuscuta pentagona*	0.55 and 0.57		McNeal et al., 2007; Neumann et al., 2020
*Cuscuta polygonorum*	0.79		McNeal et al., 2007
*Cuscuta psorothamnensis*		6I/2I + 2P	This study
*Cuscuta purpurata*	2.96		This study
*Cuscuta racemosa*	1.39	4I/2I + 2P	This study/This study
*Cuscuta reflexa*	34.73		Neumann et al., 2020
*Cuscuta rostrata*	3.98		McNeal et al., 2007
*Cuscuta sandwichiana*	1.80	2I/2I + 2P	This study/This study
*Cuscuta veatchii*	2.85	6I/2I + 2P	McNeal et al., 2007/ Ibiapino et al., 2019
OUTGROUPS			
*Calystegia sepium*	0.73		Bai et al., 2012
*Calystegia hederacea*	1.28		Guo et al., 2015
*Convolvulus arvensis*	0.65		Pustahija et al., 2013
*Convolvulus canariensis*	1.01		Suda et al., 2005
*Convolvulus cantabricus*	1.08		Pustahija et al., 2013
*Convolvulus floridus*	1.04		Suda et al., 2003
*Convolvulus perraudieri*	1.04		Suda et al. 2003
*Convolvulus scoparius*	1.04		Suda et al., 2005
*Dichondra repens*	1.57		Guo et al., 2015

* T = terminal, P = peri/centromeric, and I = interstitial.

** Ribosomal DNA sites are represented in number of sites, not in pairs.

### Flow Cytometry

A total of 11 species had their genome sizes estimated by flow cytometry, six of them here for the first time: *C. glomerata* Choisy, *C. partita* Choisy, *C. purpurata* Phil., *C. racemosa* Mart., *C. sandwichiana* Choisy, and *C. globosa* Ridl. A suspension of nuclei from shoot tips was prepared using WPB buffer (Loureiro et al., 2007). The nuclei were stained using propidium iodide and the amount of nuclear DNA was estimated using the CyFlow SL flow cytometer software (Partec, Görlitz, Germany). *Raphanus sativus* L. “Saxa” (1C = 0.53 Gbp), *Solanum lycopersicum* L. “Stupické polní rané” (1C = 0.94 Gbp), *Glycine max* (L.) Merr. “Polanka” (1C = 1.20 Gbp), and *Zea mays* L. “CE-777” (1C = 2.57 Gbp) were used as internal standards (Doležel et al., 2007). The final 2C value was based on three different measurements with 5,000 nuclei each sample, and using the equation “(Sample peak mean/Standard peak) × mean 2C DNA content of internal control (Gbp)” and the software FloMax (Partec) for data processing. The 1C value was obtained by dividing the 2C result by two.

### Reconstruction of Ancestral Chromosome Numbers

Data on chromosome numbers are summarized in Table 2. Numbers were obtained from the Chromosome Count Database (Rice et al., 2015), to which we contributed numerous counts published in several articles on the cytogenetics of the genus (e.g., García et al., 2019 and references therein). As part of our concerted efforts, these data cover at least one species for all the four subgenera, as well as the 18 sections of subgenera *Cuscuta* and *Grammica* recognized by Costea et al. (2015a). Haploid chromosome numbers were used to infer the basic ancestral numbers for each clade and the genus using ChromEvol v. 2.0 (Glick and Mayrose, 2014). To choose the model that best applies to the data set, the first run was made considering all 10 possible models of the program. Then, the model with the smallest Akaike Information Criterion (AIC) value was selected, and this model was submitted to the model adequacy test for adjustment of each selected model parameter (Rice and Mayrose, 2021). The selected model, BASE_NUM_DUPL, considered the most common chromosome number, that is, the number that appears most frequently in the phylogeny, *n* = 15, and its multiples. The parameters included in this model are the rate of increase of a single chromosome (_gainConstR), the rate of decrease of a single chromosome (_lossConstR), the rate of whole-genome duplications (polyploidy; _duplConstR), rate of transitions per base number (_baseNumberR), and the specified number of chromosomes that characterize a phylogenetic group (_baseNumber), noting that this is not the chromosome number at the root of the phylogeny (Glick and Mayrose, 2014; Rice and Mayrose, 2021).

**Table 2:** Haploid chromosome numbers (*n*) considered for character reconstruction.

Species	*n*	References
*Cuscuta approximata*	14	Guerra and García, 2004
*Cuscuta babylonica*	4	Pazy and Plitmann, 2002
*Cuscuta capitata*	10	Mehra and Vasudevan, 1972
*Cuscuta epilinum*	21	McNeal et al., 2007
*Cuscuta epithymum*	7, 8, 14, 15, 16, and 17	García and Castroviejo, 2003
*Cuscuta europaea*	7	García and Castroviejo, 2003
*Cuscuta pedicellata*	5	Pazy and Plitmann, 1995
*Cuscuta planiflora*	13 and 14	García and Castroviejo, 2003
*Cuscuta americana*	15	Neumann et al., 2020
*Cuscuta australis*	15	García and Castroviejo, 2003
*Cuscuta bonafortunae*	15	García et al., 2019
*Cuscuta brachycalyx*	15	García et al., 2019
*Cuscuta californica*	15	Neumann et al., 2020
*Cuscuta campestris*	28	García et al., 2019
*Cuscuta cephalanthi*	30	McNeal et al., 2007
*Cuscuta chapalana*	15	García et al., 2019
*Cuscuta chilensis*	15	García et al., 2019
*Cuscuta chinensis*	14	Aryavand, 1987
*Cuscuta compacta*	15	García et al., 2019
*Cuscuta coryli*	15	Fogelberg, 1938
*Cuscuta corymbosa* var. *grandiflora*	15	García et al., 2019
*Cuscuta costaricensis*	15	García et al., 2019
*Cuscuta cotijana*	15, 30	García et al., 2019, this study
*Cuscuta cuspidata*	15	Pazy and Plitmann, 1995
*Cuscuta denticulata*	15, 30	García et al., 2018
*Cuscuta desmouliniana*	15	García et al., 2019
*Cuscuta erosa*	15	García et al., 2019
*Cuscuta globosa*	45	This study
*Cuscuta glomerata*	15	García et al., 2019
*Cuscuta grandiflora*	15	García et al., 2019
*Cuscuta gronovii*	30	Love, 1982
*Cuscuta howelliana*	15	García et al., 2019
*Cuscuta indecora*	15	Ibiapino et al., 2020
*Cuscuta indecora* var. *neuropetala*	15	Fogelberg, 1938
*Cuscuta nevadensis*	15	García et al., 2018
*Cuscuta obtusiflora*	15	García et al., 2019
*Cuscuta occidentalis*	15	García et al., 2019
*Cuscuta pacifica*	15	García et al., 2019
*Cuscuta partita*	15	This study
*Cuscuta pentagona*	28	Pazy and Plitmann, 1995
*Cuscuta psorothamnensis*	30	García et al., 2018
*Cuscuta purpurata*	15	García et al., 2019
*Cuscuta racemosa*	30	García et al., 2019
*Cuscuta salina*	*ca.* 15	Pazy and Plitmann, 1995
*Cuscuta sandwichiana*	*ca.* 75	García et al., 2019
*Cuscuta sidarum*	15	García et al., 2019
*Cuscuta subinclusa*	15	García et al., 2019
*Cuscuta tinctoria*	19	Pazy and Plitmann, 1995
*Cuscuta tinctoria* var. *floribunda*	15	García et al., 2019
*Cuscuta umbrosa*	15	García et al., 2019
*Cuscuta veatchii*	30	Ibiapino et al., 2019
*Cuscuta volcanica*	15	García et al., 2019
*Cuscuta japonica*	15	Neumann et al., 2020
*Cuscuta lupuliformis*	14	McNeal et al., 2007
*Cuscuta monogyna*	15	García and Castroviejo, 2003
*Cuscuta reflexa*	16	Neumann et al., 2020
*Cuscuta africana*	14	Ibiapino et al., 2022
*Cuscuta angulata*	15	Ibiapino et al., 2022
*Cuscuta nitida*	14	Ibiapino et al., 2022

Due to the numerical chromosome variation reported in *C. epithymum* (subgenus *Cuscuta*, 2*n* = 14, 16, 28, 30, 32, and 34), *C. planiflora* (sugenus *Cuscuta*, 2*n* = 14, 26, 28, and 34), and *C. denticulata* (subgenus *Grammica*, 2*n* = 30 and 60), all the counts found were added to the ChromEvol analysis. First, we made a standard run, using the parameters given by the model adequacy test mentioned above. Then, we executed two more runs, one removing the holocentric clade (subgenus *Cuscuta*) from the analysis to test for the influence of holocentric chromosomes and intraspecific chromosome number variation. Considering the presence of holocentric and monocentric chromosomes in the genus (Fogelberg, 1938; Pazy and Plitmann, 1994), it is possible that different evolutionary models better apply for different clades (Márquez-Corro et al., 2019). In the second additional run, we fixed *n* = 15 to the root, because this is the basic number proposed for *Cuscuta* from cytogenetic data (Pazy and Plitmann, 2002) and was supported by the data compilation produced in this work. The results were plotted in R using the ChromEvol functions as described in Cusimano et al. (2012).

An additional reconstruction of the ancestral chromosome number was performed in Mesquite version 2.75, using maximum likelihood (Maddison and Maddison, 2018) to compare results with those originated from ChromEvol. However, in Mesquite, the haploid chromosome numbers were categorized into nine states: *n* = 4 (coded as 0), *n* = 5 (1), *n* = 7 (2), *n* = 10 (3), *n* = 13 (4), *n* = 14 (5), *n* = 15 (6), *n* = 16 (7), *n* = 19 (8), and all polyploids from *n* = 21 to *n* = 75 (9). Additionally, we compared the results to the inference of the ancestral state of this character made along the branches using PastML (Ishikawa et al., 2019).^1^ We applied the JOINT (highest likelihood) method. As the model assume only one state per sample, we used for *C. epithymum and C. planiflora* the cytotypes analyzed in the present work, *n* = 14, and for *C. denticulata*, *n* = 15.

### Reconstruction of Genome Sizes

Genome size estimations for six species were newly obtained for this paper in addition to new assessments for five species with previously published data. The reconstruction was performed for 28 *Cuscuta* species in total (Table 1), three of subgenus *Cuscuta,* 20 of subgenus *Grammica*, and five of subgenus *Monogynella.* This trait was analyzed as a continuous character in the phytools package (Revell, 2012). Thirty-three taxa that lacked GS information were excluded from our original tree using the ape package (Paradis et al., 2004) and we included three additional species with known genome size but unknown chromosome numbers (*C. rostrata* Engelm. & A. Gray, *C. polygonorum* Engelm., and *C. exaltata* Engelm.). Both phytools and ape packages were implemented in R (R Core Team, 2020). For this analysis, we considered the value of 1C in Gbp, and for species with two or more genome sizes published, an average was made between the values. In addition, for comparative purposes, a reconstruction of the genome size was also performed in Mesquite using the implemented maximum parsimony analysis.

To address whether the inclusion of outgroups changed significantly the results of the previous analyses, we performed three additional reconstructions of the ancestral genome size of *Cuscuta*. Each one included as outgroup candidates of Convolvulaceae with genome size data available of the two sister groups resolved by Stefanović and Olmstead (2004) as the closest relatives of *Cuscuta*. One of the analyses used two species of *Calystegia* R.Br. and six of *Convolvulus* L. (Convolvuleae, clade 1), another one *Dichondra repens* J.R.Forst. & G.Forst. (Dichondreae, clade 2), and the third one all of them.

### Reconstruction of rDNA Ancestral Positions

The reconstruction of ancestral number and positions of the 5S and 35S rDNA sites were performed using Mesquite version 2.75 (Maddison and Maddison, 2018) on the 18 species for which rDNA information was available, 10 of them newly generated for this paper. Both the number and position of sites were transformed into categorical data (discrete characters): centromeric/pericentromeric position, interstitial, terminal/subterminal, and “mix” (when more than one of the previous conditions occurs in the same karyotype) as proposed by García et al. (2017). For the number of 5S sites, the characters were categorized as 1, 2, 3, 5, or 18 pairs. The number of 35S sites was categorized as 1, 2, 5, or 15 pairs of sites. Ancestral character states were inferred using maximum likelihood (Vaio et al., 2013). Due to the inconclusive results obtained in the rDNA sites number reconstruction using Mesquite, a second reconstruction was conducted using the Bayesian Binary MCMC (BBM) tool (Ali et al., 2012) implemented in the software Reconstruct Ancestral State in Phylogenies—RASP 4.2 (Yu et al., 2015, 2020) using the default parameters.

## Results

### Phylogenetic Reconstruction

In total, 57 species of *Cuscuta* with DNA sequences and cytogenetic data available were sampled for the phylogenetic reconstruction, representing approximately 30% of the *ca.* 200 known species of the genus. The four subgenera were recovered as monophyletic each. Subgenus *Monogynella* was represented by four out of 15 species (26.67%), *Cuscuta* by eight out of 22 (36.36%), *Pachystigma* by three out of five (60%), and *Grammica* by 42 out of 150 (28.6%). The phylogenetic relationships obtained in this study were consistent with those based on a larger dataset reported by García et al. (2014), in which each of the four subgenera was strongly supported as monophyletic, with subgenus *Monogynella* as sister to the rest. The trees resolved the same relationships between the sections of subgenera *Cuscuta* and *Grammica* as those obtained by García et al. (2014; Supplementary Figure 1). The phylogenetic position of *C. globosa* was resolved as a member of section *Gracillimae* (clade N). Based solely on the description of the type, this species had previously been included in section *Racemosae* (clade C; Costea et al., 2015a). Here, it is placed in section *Gracillimae* based on molecular data, in agreement with the morphological features studied in the type and our new collections of this species.

### Chromosome Number, rDNA Site, and Genome Size Variation in *Cuscuta*

Most *Cuscuta* species with published chromosome number are diploids with up to 2*n* = 38 (40 species plus two varieties, Table 2). Another 11 species are polyploids, mostly with 2*n* = 60. Of the species included in this study, four of them are known to have diploid and tetraploid populations: *C. planiflora*, *C. epithymum*, *C. cotijana* (2*n* = 60, a new cytotype; Supplementary Figure 2), and *C. denticulata*. Two new counts were included in this work, *C. partita* (2*n* = 30) and *C. globosa* (2*n* = 90; Supplementary Figure 2). The majority of polyploids belongs to subgenus *Grammica*, with the exception of some tetraploids and hexaploids of subgenus *Cuscuta*, such as *C. approximata* (2*n* = 28) or *C. epilinum* Weihe (2*n* = 42), considering a lower basic number for this subgenus (see below). The smallest number found was 2*n* = 8 in *C. babylonica* Aucher ex Choisy (*Cuscuta*), while the largest number was 2*n* = 150 in *C. sandwichiana* (*Grammica*). *Cuscuta epithymum* (*Cuscuta*) presents numerical intraspecific variation with 2*n* = 14, 16, 28, 30, 32, and 34.

**Figure 1:**
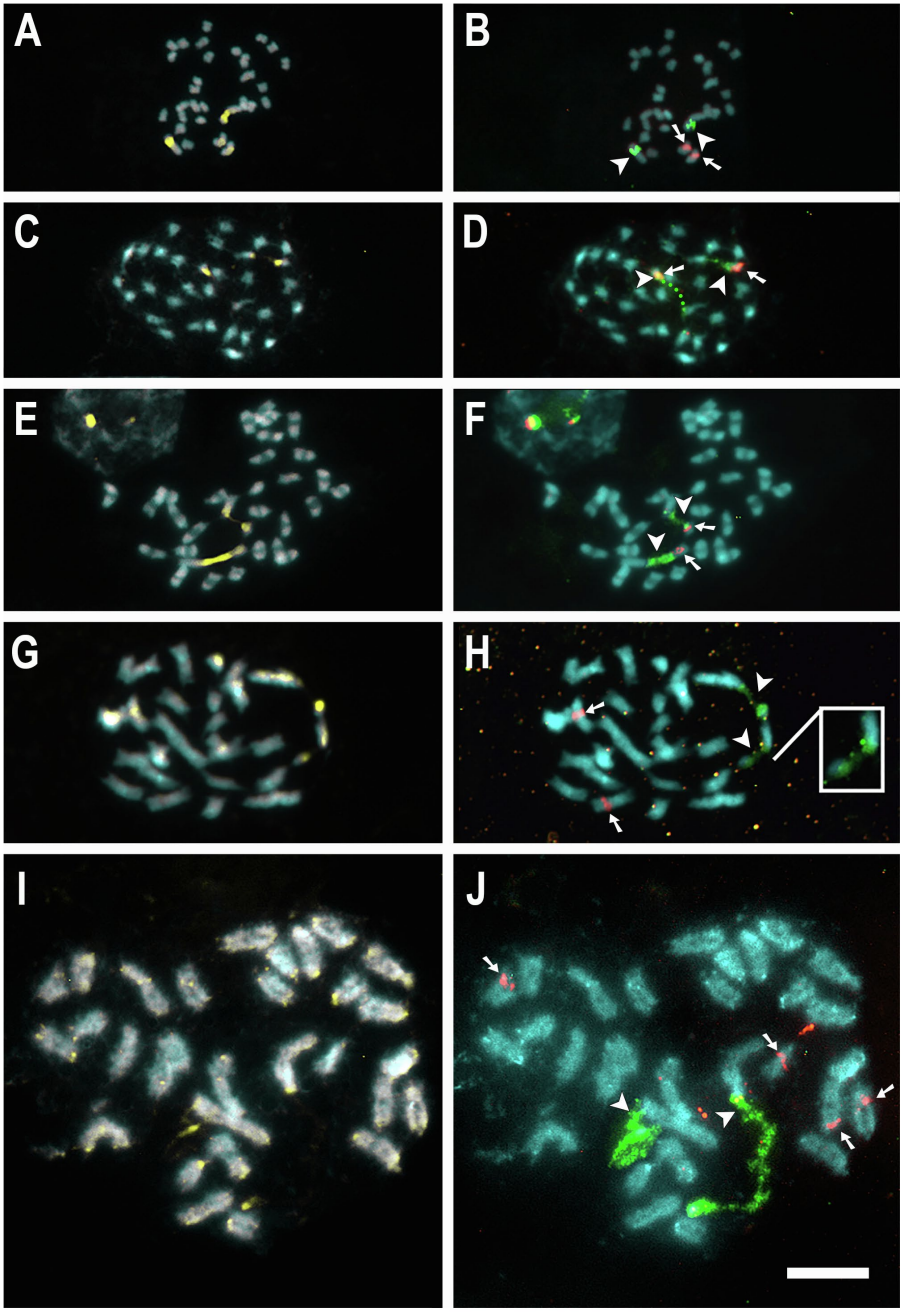
Mitotic metaphases of diploids with karyotypes 2*n* = 30. *C. australis* **(A,B)**, *C. howelliana* **(C,D)**, *C. partita* **(E,F)**, *C. glomerata* **(G,H)**, and *C. epithymum* **(I,J)** stained with CMA (yellow) and DAPI (blue) in **A, C, E, G,** and **I**, and with FISH of 5S (red) and 35S (green) rDNA in **B, D, F, H,** and **J**. Arrowheads indicate 35S (green) and arrows indicate 5S (red) rDNA sites. Insets show weak signals in higher contrast. Bar in **J** represents 10 μm; all images at the same magnification.

**Figure 2:**
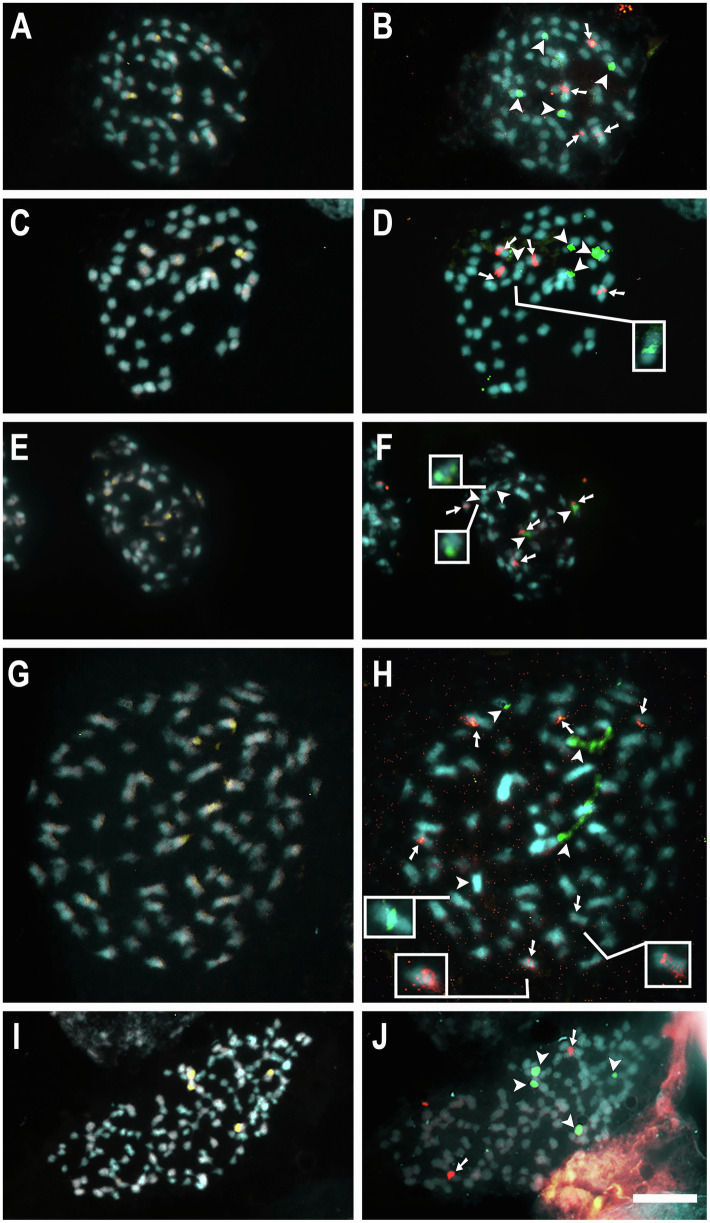
Mitotic metaphases of the polyploids *C. campestris* 2*n* = 56 **(A,B)**, *C. racemosa* 2*n* = 60 **(C,D)**, *C. psorothamnensis* 2*n* = 60 **(E,F)**, *C. globosa* 2*n* = 90 **(G,H)**, and *C. sandwichiana* 2*n* = 150 **(I,J)** stained with CMA (yellow) and DAPI (blue) in **A, C, E, G,** and **I**, and with FISH of 5S (red) and 35S (green) rDNA in **B, D, F, H,** and **J**. Arrowheads indicate 35S (green) and arrows indicate 5S (red) rDNA sites. Insets show weak signals in higher contrast. Bar in **J** represents 10 μm.

For seven species, 5S and 35S rDNA site number and location were previously published (Table 1; Guerra and García, 2004; Ibiapino et al., 2019, 2020, 2022). New rDNA data were obtained for 10 additional species, *C. australis* (2*n* = 30), *C. campestris* Yunck. (2*n* = 56), *C. epithymum* (cytotype with 2*n* = 30), *C. howelliana* P. Rubtzoff (2*n* = 30), *C. partita* (2*n* = 30), *C. psorothamnensis* Stefanović, M. A. García & Costea (2*n* = 60), *C. racemosa* (2*n* = 60), *C. sandwichiana* (2*n* = 150), *C. globosa* (2*n* = 90), and *C. glomerata* (2*n* = 30). All rDNA sites in *Cuscuta* were colocalized with CMA^+^ bands. Most species presented at least one pair of CMA^+^/DAPI^−^ bands colocalized with nucleolus organizer regions (NOR) in proximal regions. Interstitial bands, when present, were weaker and smaller. Only in the holocentric *C. epithymum* did these bands occur in the terminal regions and were present in most chromosomes (Figures 1, 2). Most species showed only one pair of 5S and one pair of 35S rDNA sites. Usually, 5S sites occurred in interstitial regions, while 35S sites in pericentromeric regions, such as in *C. australis*, *C. howelliana*, *C. partita*, and *C. glomerata* (Figure 1). When more than one pair of 5S rDNA was present, these sites were also in interstitial regions, whereas when there were more than one pair of 35S, the extra pairs were interstitial, as in *C. campestris*, *C. racemosa*, *C. psorothamnensis*, *C. globosa,* and *C. sandwichiana* (Figure 2), or terminally located, as in *C. epithymum* (Figure 1). The largest number of rDNA sites in *Cuscuta* species was observed in *C. monogyna*, with approximately 18 pairs of 5S and 15 pairs of 35S rDNA. Information on the number and distribution of the rDNA in *Cuscuta* can be found in Table 1.

**Figure 3:**
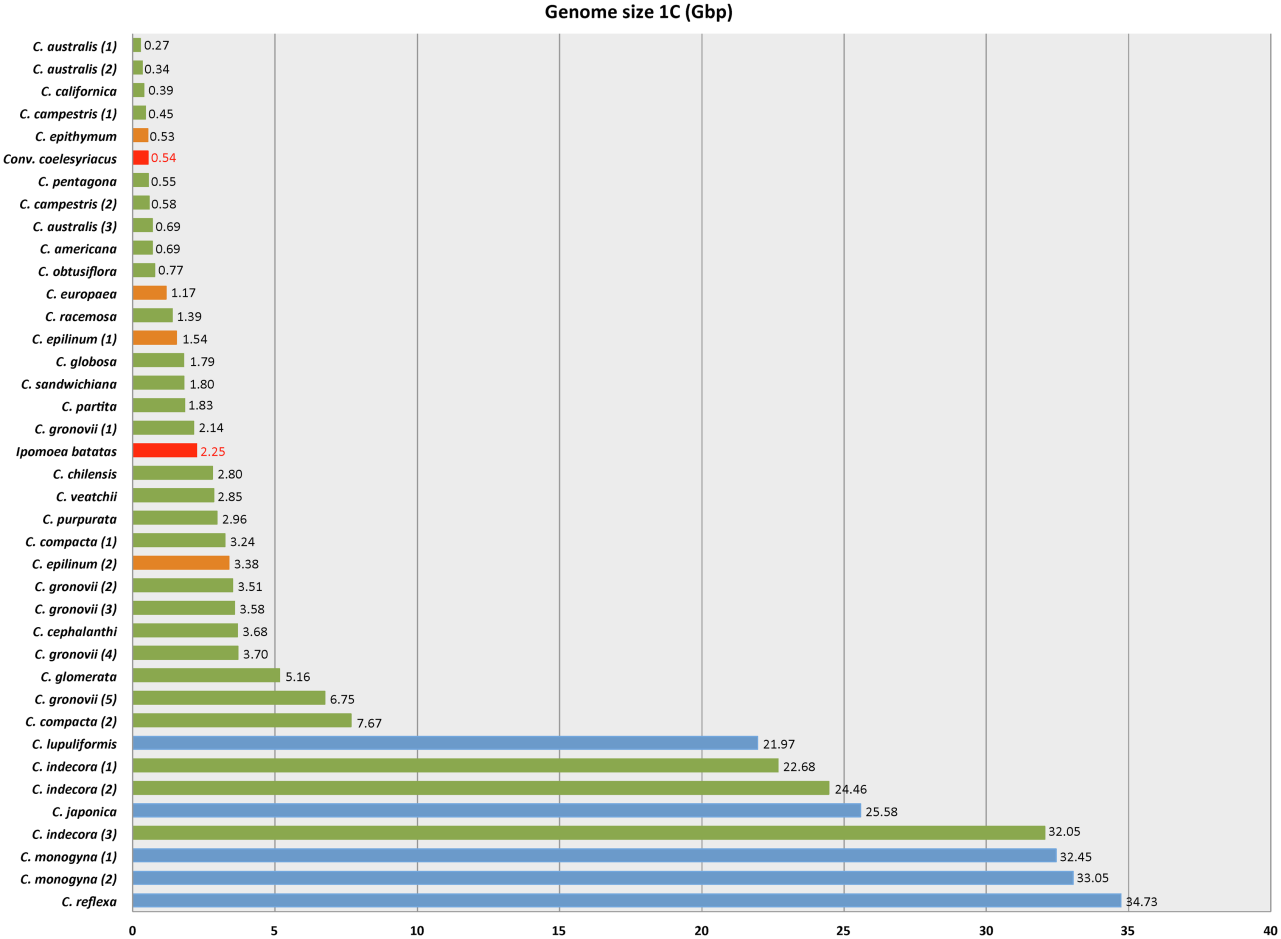
Genome size variation in *Cuscuta* based on the data in Table 2. Red bars indicate the lowest and highest known genome sizes in other Convolvulaceae, as reported in the Plant DNA C-values Database (https://cvalues.science.kew.org/). Color bars for *Cuscuta* indicate the subgenera: blue for *Monogynella*, green for *Grammica,* and orange for subgenus *Cuscuta.*

As for genome size, *Cuscuta* species varied from 1C = 0.27 Gbp in *C. australis* (2*n* = 30) to 1C = 34.73 Gbp in *C. reflexa* (2*n* = 32), both diploids. This variation represents the lowest and highest genome sizes known for Convolvulaceae (Figure 3). Some species showed infraspecific variation in genome size, such as *C. gronovii* Wild. ex Roem. & Schult., with five different values reported, ranging from 1C = 2.14 Gbp to 1C = 6.75 Gbp (Table 1).

### Ancestral Character State Reconstructions

The chromosome number reconstruction performed in ChromEvol with the best model, BASE_NUM_DUPL, indicated *n* = 7 as the basic ancestral number in *Cuscuta* (Supplementary Figure 3). This model considers five parameters, the rates of gains and losses of single chromosomes, duplications, in addition to considering a specific chromosome number that characterizes a phylogenetically close group, and the number variation rate. Based on this model, the variation in chromosome number in *Cuscuta* is most often related to duplication events [with a probability of frequency (*f* = 9.2)], followed by chromosome gains (*f* = 8.3) and losses (*f* = 7). The number *n* = 15 was indicated as ancestor of the subgenera *Grammica* and *Monogynella*. The ancestral number of subgenus *Cuscuta* was *n* = 7, and *Pachystigma* had *n* = 14, with 50% probability, but *n* = 7 was also very likely, with 40% probability (Supplementary Figure 3).

**Figure 4:**
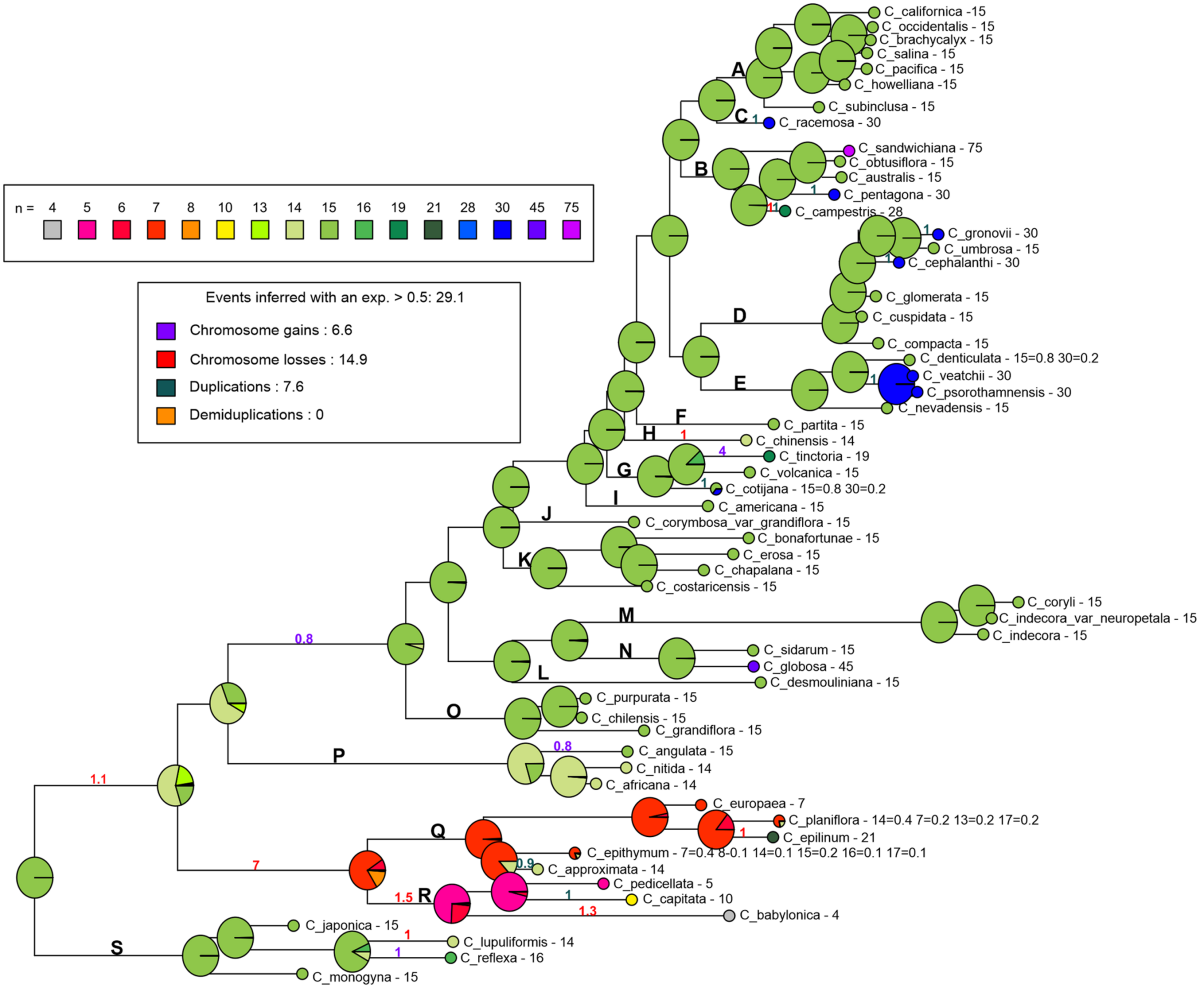
Reconstruction of the chromosome number evolution in *Cuscuta* with the BASE_NUM_DUPL model and the number *n* = 15 fixed in the base. The pie charts at nodes represent the probability of each inferred chromosome number, the numbers along the branches represent the probability of frequencies of the inferred events (gains, losses, duplications, and demiduplications). The bold letters represent the sections described by García et al. (2014): section S (subgenus *Monogynella*), sections R and Q (subgenus *Cuscuta*), section P (subgenus *Pachystigma*), and sections A–O (subgenus *Grammica*).

The holocentric clade (subgenus *Cuscuta*) includes species with intraspecific numerical variation. To test if its holocentric nature and high chromosome number variation influenced the analysis, we ran ChromEvol without the holocentric clade, following the same parameters described above (Supplementary Figure 4). The basic number in this analysis was *n* = 15 (*x* = 15), again with chromosome duplication (*f* = 7), followed by chromosome gains (*f* = 5.6) and chromosome losses (*f* = 3.7) as the main evolutive events. The reconstructed ancestral number for the remaining three subgenera was conserved as *n* = 15 (Supplementary Figure 4). Therefore, we repeated ChromEvol analysis fixing *n* = 15 at the base of the genus (Figure 4). In this scenario, numerical changes were mainly due to chromosome losses (*f* = 14.9), followed by duplications (*f* = 7.6) and chromosome gains (*f* = 6.6). The reconstructed ancestral numbers for *Grammica* and *Monogynella* were also *n* = 15. In subgenus *Cuscuta*, the basic number was *n* = 7 and the basic number for *Pachystigma* was *n* = 14 (Figure 4).

Mesquite and PastML also recovered *n* = 15 as the basic chromosome number for the whole genus. Furthermore, the maximum likelihood analysis suggested *n* = 15 for the subgenera *Monogynella* and *Grammica*. For subgenus *Pachystigma*, the most likely basic number reconstructed was *n* = 14 (with 50% probability), but *n* = 15 was also likely (with 43% probability). Only subgenus *Cuscuta* had three possible basic numbers depending on the analysis. The Mesquite analysis retrieved *n* = 7 and 14 as the most likely, each with approximately 31% probability (Supplementary Figure 5). The PastML JOINT approach reconstructed *n* = 15 as the ancestral number for both the entire genus and each of its four subgenera (data not shown).

**Figure 5:**
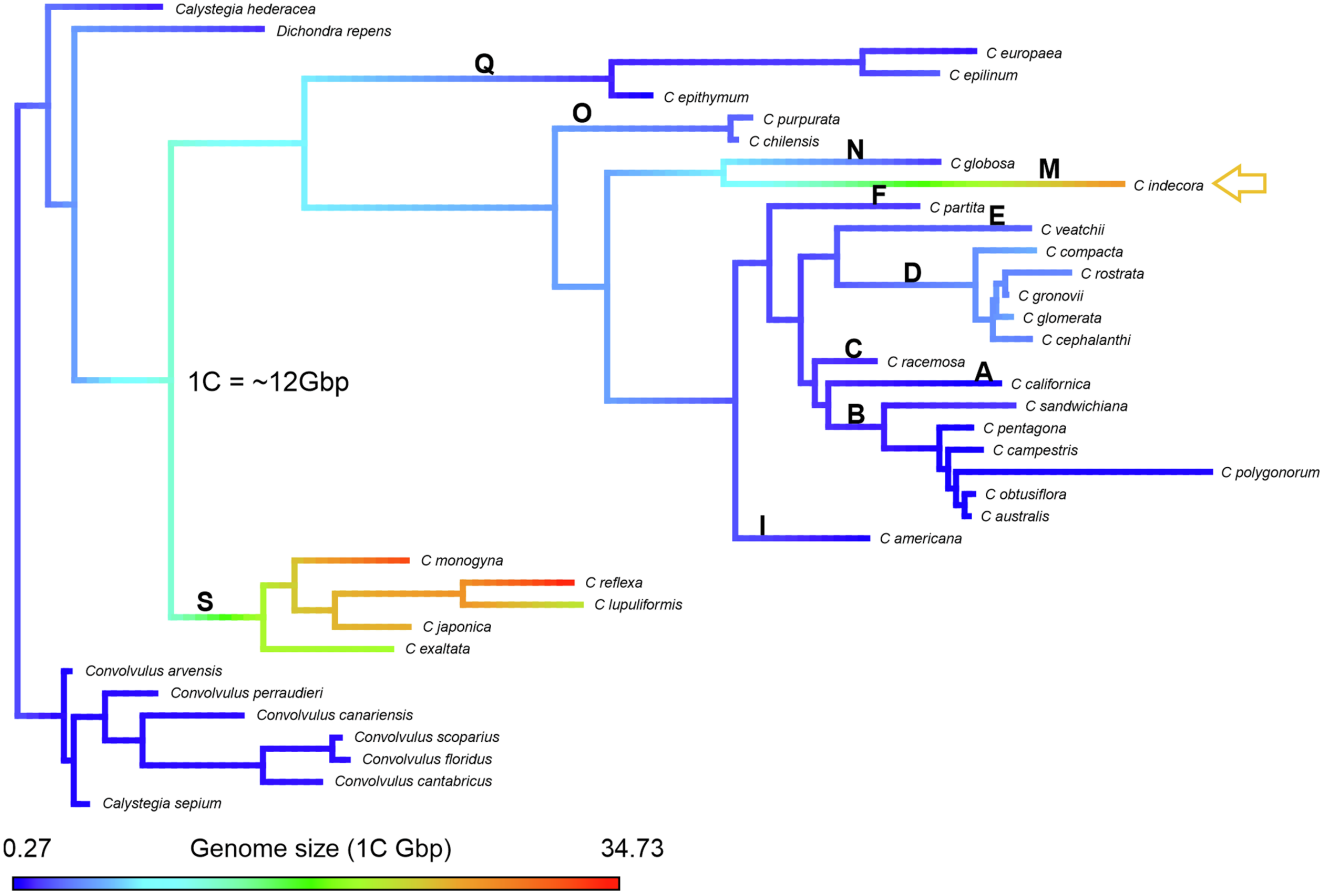
Reconstruction of the genome size evolution *via* Phytools in R. The variation is shown in a color scale: the largest genomes in shades of red and the smallest genomes in shades of blue. The yellow arrow in the reconstruction indicates *C. indecora*, a species of the subgenus *Grammica* in which there was a massive expansion of the genome. The bold letters represent the sections described by García et al. (2014): section S (subgenus *Monogynella*), sections R and Q (subgenus *Cuscuta*), section P (subgenus *Pachystigma*), and sections A–O (subgenus *Grammica*).

Considering the smallest genome of 1C = 0.27 Gbp and the largest of 1C = 34.73 Gbp, the reconstructions of ancestral genome size made with phytools suggested that the ancestral genome of *Cuscuta* would be of intermediate size, approximately 20 Gbp without outgroups (Supplementary Figure 6), and 1C = 12 Gbp, including available outgroups. The results obtained using *Dichondra* or *Calystegia* and *Convolvulus* or these three genera as outgroup were similar (14, 12, and 12 Gbp, respectively; Supplementary Figure 6). Genome size increased in subgenus *Monogynella*, while it decreased in the other subgenera. Only *C. indecora* showed a massive expansion of genome size within subgenus *Grammica* (Figure 5), what was also observed with Mesquite (data not shown). Although there is no estimation available for subgenus *Pachystigma,* the long chromosomes of their bimodal karyotypes also suggest an increase in genome size, especially for *C. angulata* (Ibiapino et al., 2022).

**Figure 6:**
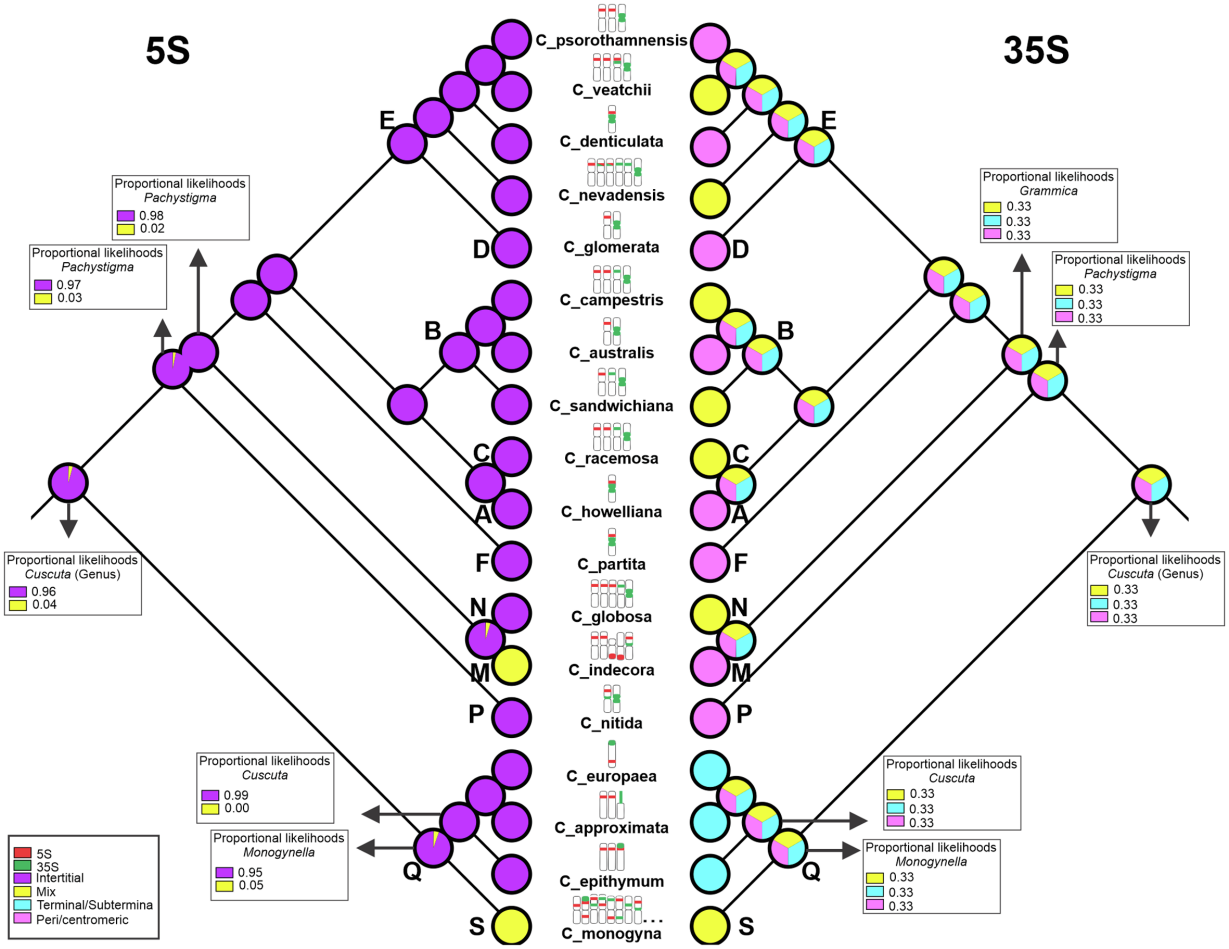
Reconstruction of the position of the 5S (left) and 35S (right) rDNA sites. In subgenus *Monogynella,* there is an increase in the diversity of positions in which the rDNA sites were found, suggesting that the “mix” condition is derived. In *C. monogyna,* not all pairs are represented because it is a species that has more than 30 rDNA sites, but all observed patterns are outlined. The bold letters represent the sections described by García et al. (2014): section S (subgenus *Monogynella*), sections R and Q (subgenus *Cuscuta*), section P (subgenus *Pachystigma*), and sections A–O (subgenus *Grammica*).

The reconstruction of the ancestral number of rDNA sites made by Mesquite was inconclusive (data not shown). The analysis performed using RASP reconstructed 18 pairs of sites as ancestral for 5S rDNA and 15 pairs for 35S rDNA, but it is probably due to the presence of numerous sites in *C. monogyna* (Supplementary Figure 7). The reconstruction of rDNA site positions indicated that the ancestral position of the 5S rDNA was likely interstitial. The reconstructed position of the 35S rDNA site was inconclusive, with *C. monogyna* showing terminal and interstitial sites. Interstitial 5S rDNA sites were maintained throughout the genus. Only in *C. indecora* additional sites in terminal positions appeared. The 35S rDNA was reconstructed as terminal in the subgenus *Cuscuta* and peri/centromeric in *Grammica*. In this latter subgenus, species with only a pair of 35S sites had them always in peri/centromeric position. When more than one pair of 35S rDNA was present, the extra sites were inferred to have originated in interstitial positions (Figure 6).

## Discussion

### Evolution of Chromosome Number in *Cuscuta* and the Uniqueness of the Holocentric Clade

Chromosome number variation across the entire genus *Cuscuta* was almost 19-fold between 2*n* = 8 in *C. babylonica* and 2*n* = 150 in *C. sandwichiana* (Pazy and Plitmann, 2002; García et al., 2019). The subgenus *Cuscuta* alone showed a variation of over 5-fold (from 2*n* = 8 to 2*n* = 42). The variation within this subgenus is also associated with intraspecific numerical variation, found in *C. epithymum* (2*n* = 14, 16, 28, 30, 32, and 34) and *C. planiflora* (2*n* = 14, 26, 28, and 34; García and Castroviejo, 2003). Subgenus *Grammica* species diversity was less represented with only ca. 30% of its ca. 150 species (Stefanović et al., 2007; Costea et al., 2015a). However, the variation in chromosome numbers found was similar to that observed in subgenus *Cuscuta*, just over 5-fold (2*n* = 28 to 2*n* = 150). In this case, most of the variation is attributable to auto- or allopolyploidy and only a few species have a chromosome number that is not *n* = 15 or a multiple thereof. The additional numbers can be explained by ascending or descending dysploidy. The relative low proportion of species studied suggests that the variation in this subgenus may be underestimated. Hybrid speciation was frequent in subgenus *Grammica* (e.g., Stefanović and Costea, 2008; Costea and Stefanović, 2010; García et al., 2014; Costea et al., 2015b) and a more detailed sampling will probably reveal additional cases of both auto- and allopolyploidy, as was recently reported for the small section *Denticulatae* (clade E; García et al., 2018).

In subgenera *Monogynella* and *Pachystigma*, no large variation in chromosome number was found. In this work, *Monogynella* is represented by five of its 15 species and the most common chromosome numbers found were 2*n* = 28, 30, and 32. Two additional species of the subgenus without sequence data were reported to have 2*n* = 32 chromosomes, *C. gigantea* Griff. (Khatoon and Ali, 1993) and *C. sharmanum* Mujerjee & P. K. Bhattach. (Mukerjee and Bhattacharya, 1970). Only *C. reflexa* has been reported to have a great intraspecific variation of chromosome numbers with triploid, tetraploid, and dysploid or aneuploid individuals (Kaul and Bhan, 1977). The small subgenus *Pachystigma*, represented by three of its total of five species (60%), has 2*n* = 28 and 30. The species of this subgenus have bimodal karyotypes; however, both the number of large and small pairs and the distribution of heterochromatic bands are quite variable (Ibiapino et al., 2022).

We used ChromEvol to reconstruct the basic ancestral chromosome number, which indicated the BASE_NUM_DUPL model as the best for our dataset. With this model, the basic ancestral number for *Cuscuta* was *x* = 7, as previously suggested by Fogelberg (1938) and García and Castroviejo (2003). However, most of the chromosome numbers reported in *Cuscuta* are multiples of 15, because 32 species (55%) are diploids with 2*n* = 30 and, among polyploids, 2*n* = 60 is the most frequent number. The highest numbers reported are 2*n* = 90 and 150, which are also multiples of 15. Additional analyses of both Mesquite and Past ML corroborated the number *x* = 15 at the base of the genus *Cuscuta*.

According to the Chromosome Count Data Base (Rice et al., 2015), the most frequent chromosome number in other Convolvulaceae is *n* = 15. Because of the accelerated rates of sequence evolution in *Cuscuta*, the extant sister group of the genus within Convolvulaceae could not be ascertained, but at least two nonparasitic lineages diverged before *Cuscuta* (Stefanović and Olmstead, 2004). Chromosome numbers in these lineages are known for a couple of genera in tribe Cardiochlamyeae: *n* = 13 for *Poranopsis* Roberty and *n* = 14 for *Dinetus* Sweet. Among more closely related lineages chromosome numbers are mostly *n* = 14 or 15, but some genera show greater diversity in chromosome numbers such as *Convolvulus* L. (*n* = 9–30), *Merremia* Endl. (*n* = 7–29), or *Ipomoea* L. (*n* = 14–45). Altogether, it is unlikely that, in *Cuscuta*, the ancestral number has reduced to *x* = 7, followed by independent chromosome duplications and gains in *Monogynella* and *Pachystigma* + *Grammica*. Therefore, we consider *x* = 15 more likely for the genus *Cuscuta*.

One reason for the inference of *x* = 7 for the genus is probably the presence of lower numbers in the subgenus *Cuscuta*. However, this subgenus is exclusively holocentric, and this chromosome type may go through karyotypic changes that are different than those in the other subgenera. Chromosome fusion and fission events can be favored in this karyotype type, as they have a diffuse kinetochore, which facilitates these types of rearrangements (Mandrioli and Manicardi, 2020). In groups where different evolutionary dynamics occur, it is necessary to consider clade specific models, that is, different parts of the phylogeny evolving according to different transition patterns of changes in chromosome numbers (Mayrose and Lysak, 2021). Márquez-Corro et al. (2019) used different methodological approaches to identify diverse patterns of chromosomal evolution in some clades of Cyperaceae. In that case, both a complete tree and subtrees were analyzed, suggesting several evolutionary model transitions in the entire phylogeny of the family. This type of analysis is particularly relevant when applied to the study of clades containing species with holocentric chromosomes, whose karyotypes can exhibit heterogeneous evolution modes. Therefore, we removed the holocentric clade to evaluate the reconstruction. In this analysis, the basic number *x* = 15 was retrieved for both the genus as a whole, and in each of the remaining subgenera, with duplication events as the most frequent resulting in the formation of polyploids. This corroborated the idea that the evolutionary dynamics of holocentrics are indeed different, and it exerted a large influence on the reconstruction of chromosome numbers in *Cuscuta.*

We therefore considered *n* = 15 fixed at the base of the genus *Cuscuta* as the best model to explain the evolution of chromosome numbers of this genus. In this model, seven chromosome losses occurred leading to *x* = 7 after the transition from monocentric to holocentric chromosomes in the subgenus *Cuscuta*. For the holocentric species, the chromosome numbers found were 2*n* = 8, 10, 14, 16, 18, 20, 26, 28, 30, 32, 34, and 42. Among the three sections of subgenus *Cuscuta* recognized by Costea et al. (2015a), the lowest chromosome number, 2*n* = 8, was found in *C. babylonica*, the only species of the monotypic section *Babylonicae*. This section is generally recovered as sister to section *Epistigma*, a group of five, mostly Asian species with chromosome numbers known in *C. pedicellata* Ledeb. and *C. pulchella* Engelm., both with 2*n* = 10, and *C. capitata* Roxb., probably a tetraploid with 2*n* = 20. The lineage of sections *Epistigma* and *Babylonicae* may have reduced their chromosome number by descending dysploidy through chromosome fusions. In this lineage, the biggest chromosomes are in *C. babylonica*, which shows the lowest chromosome number.

In the sister lineage, section *Cuscuta* of the subgenus *Cuscuta*, diploid species have at least 2*n* = 14 chromosomes, several species such as *C. approximata* and *C. palaestina* Boiss. are tetraploids (2*n* = 28), and *C. epilinum* is a hexaploid (2*n* = 42). Other diploids, not included in our analyses, have higher chromosome numbers, such as 2*n* = 18 and 20 reported for *C. nivea* M.A. García (García, 2001). Two species, *C. epithymum* and *C. planiflora*, are known to have diploid and polyploid populations with further variation in chromosome numbers that may have been generated from duplication and ascending dysploidy events. In *C. epithymum* (2*n* = 14, 16, 28, 30, 32, and 34), there are diploid and tetraploid cytotypes with both bimodal and symmetrical karyotypes. Some cytotypes with 2*n* = 14 and 2*n* = 32 are bimodal, whereas others with 2*n* = 16 and 2*n* = 34 are symmetrical (García and Castroviejo, 2003; García et al., 2019), suggesting that chromosome fusion or fission events, together with polyploidy, engender this numerical variation. The cytotypes with asymmetrical karyotypes in *C. epithymum* and the other species of subgenus *Cuscuta* have the active NORs located in the longest chromosomes and with hematoxylin staining they are observed associated to the nucleoles. In *C. planiflora* (2*n* = 14, 26, 28, and 34), the populations with 2*n* = 34 have an asymmetrical karyotype that together with morphological features indicate that it is an allopolyploid (García, 2001). Both, *C. epithymum* and *C. planiflora* are taxonomically difficult as revealed by the high number of infraspecific taxa that have been described (García and Martín, 2007), and their variation in chromosome numbers and karyotypes may indicate cryptic diversity in these species’ complexes.

### Genome Size Variation in *Cuscuta* Is Extreme and Is Reflected in Chromosome Sizes

Genome size data for 28 species of *Cuscuta* (Table 1, Figure 5) revealed a 128-fold variation between the smallest (1C = 0.27 Gbp in *C. australis*) and the largest genome (1C = 34.73 Gbp in *C. reflexa*). This tremendous variation in *Cuscuta* does not seem to be mainly caused by polyploidy events, despite its high frequency in the genus (García et al., 2019; Ibiapino et al., 2019). Polyploids with higher chromosome numbers, such as *C. globosa* (2*n* = 90) and *C. sandwichiana* (2*n* = 150), both belonging to subgenus *Grammica*, had small genome sizes, 1C = 1.79 Gbp and 1C = 1.8 Gbp, respectively. *Cuscuta*, therefore, fits with the general trend of genome downsizing of polyploids observed in angiosperms (Leitch and Leitch, 2008). Subsequent genome downsizing in polyploids is also known from other parasitic lineages (e.g., in *Orobanche*; Weiss-Schneeweiss et al., 2006).

It is remarkable that the largest genome sizes are found in diploid species such as *C. lupuliformis* Krock. (2*n* = 28) and *C. reflexa* (2*n* = 32), both belonging to subgenus *Monogynella*, with 1C = 21.97 Gbp and 1C = 34.73 Gbp, respectively. Species such as *C. monogyna* (1C = 33.05 Gbp, subgenus *Monogynella*) and *C. indecora* (1C = 24.46 Gbp, subgenus *Grammica*), both diploids, have numerous heterochromatic bands along their chromosomes, all these bands co-localizing with repetitive DNA such as 5S and 35S rDNA or satellite DNAs, indicating that the amplification of repetitive sequences in heterochromatin is involved (Ibiapino et al., 2020; Naumann et al., 2020). However, there is also an accumulation of heterochromatic bands in subgenus *Cuscuta*, but there is no drastic increase in genome size probably because of the reduction in the number of chromosomes associated with the transition to holocentric chromosomes. In the diploid *C. europaea* L., for example, the genome size is, on average, 1C = 1.11 Gbp (McNeal et al., 2007; Neumann et al., 2020). This species has satellite DNAs that occupy a large extension of all 14 chromosomes, which varies in sizes from 2.76 to 6.70 μm, approximately (estimated measurements based on mitotic metaphases reported in Oliveira et al., 2020). In subgenus *Pachystigma*, the differential accumulation of repeats in just a few chromosomes led to the appearance of bimodal karyotypes. *Cuscuta nitida* E. Mey. ex Choisy (2*n* = 28), for example, has an accumulation of different classes of repetitive DNA in only two chromosome pairs, which are, in average, 12.34 and 8.19 μm long, compared to 2.67 μm of the smallest pairs which is not enriched with repetitive DNA (Ibiapino et al., 2022). Thus, the accumulation of repetitive DNA can lead to an increase in chromosomes and, consequently, to an increase in genome size, especially in subgenus *Monogynella*.

Although genome size has been estimated for just a few species, the relative chromosome size may be an indirect indicator of genome size if we compare species with the same ploidy level. The smallest genome size in the genus estimated for the diploid *C. australis* (2*n* = 30; 1C = 0.27 Gbp) correlates well with the small chromosome size observed in mitotic metaphases (Figures 1A,B). Other diploids with the same chromosome number and similar chromosome size probably have a similar genome size. Such is the case of *C. desmouliniana* Yunck. (García et al., 2019) for which no genome size has been measured, but the size of chromosomes is similar or even smaller than in *C. australis*.

In the phylogeny of *Cuscuta*, some clades include species with noticeable differences in chromosome size. In subgenus *Pachystigma,* the number of large chromosomes varies from two pairs in *C. nitida* to five pairs in *C. angulata* suggesting a great difference in genome size within the subgenus. Further analysis of the two unsampled species from Eastern South Africa might reveal an even greater variation. Subgenus *Grammica*, accounting for ca. 70% of the *Cuscuta* species diversity (Stefanović et al., 2007), shows several clades with significant differences in chromosome and genome size between closely related species. A remarkable example is the strongly supported clade encompassing sections *Umbellatae*, *Indecorae*, and *Gracillimae* (clades L, M, N; García et al., 2014; Costea et al., 2015a), which has species with very small (*C. desmouliniana*, clade L), intermediate (*C. sidarum* and *C. globosa*, clade M), and large chromosomes (*C. coryli* Engelm. and *C. indecora*, clade M). The branch leading to species in sect. *Indecorae* is noticeably longer compared to others in the subgenus, showing an increase in mutation rates together with the increase of the genome size.

Although not as evident as in subgenus *Pachystigma*, some sections of subgenus *Grammica* (Costea et al., 2015a) appear to have a prevalence of asymmetrical karyotypes suggesting differential accumulation of DNA is some chromosome pairs (García et al., 2019) and probably to an increase in genome size. In section *Ceratophorae* (clade K; Costea et al., 2011), from which only diploid species are known, *C. costaricensis* Yunck. has one pair of chromosomes noticeably longer than the others, whereas in *C. bonafortunae* Costea & I. García or *C. erosa* Yunck. about half are long and half are shorter. Similar karyotypes have been documented in allopolyploids such as *C. veatchii*, but in these cases, the asymmetry is a consequence of the subgenomes of the two diploid parents with differences in chromosome size (Ibiapino et al., 2019).

Whereas some sections of subgenus *Grammica* show a tendency to increase genome size, others have undergone a significant reduction. The expansion and diversification of the genus in North America resulted in two lineages (Stefanović et al., 2007; García et al., 2014) having opposing directions in the evolution of genome size. The lineage of sections *Oxycarpae* (clade D) and *Denticulatae* (clade E) shows genome upsizing, which is especially evident in the former, including species with the biggest genomes in subgenus *Grammica* except for sect. *Indecorae*. On the contrary, the lineage that includes sections *Californicae* (clade A) and *Cleistogrammica* (clade B) has the smallest genomes not only in *Cuscuta* but also in Convolvulaceae. The only species of section *Racemosae* (clade C) with known karyotype and genome is *C. racemosa* (2*n* = 60; 1C = 1.39), having a relatively small genome compared to other tetraploids. Further sampling in this section will reveal whether the dispersal to South America before the diversification of this clade was accompanied by a significant variation in genome size.

Table 1 also summarizes the great intraspecific variation in the genome size of species of sections *Oxycarpae* and *Indecorae* (Costea et al., 2015a). Within *Oxycarpae*, differences such as in *C. compacta* (1C = 3.24 and 7.67) suggest that there might be diploid and tetraploid populations. In *C. gronovii*, all the chromosome counts are 2*n* = 60; however, the genome size reported for the species ranges from 1C = 2.14 and 1C = 6.75 Gbp. Taxonomy of the section in general and of *C. gronovii* in particular is difficult and these differences might be explained by species identification mistakes or might reflect the morphological diversity of the species, for which several varieties have been described (Yuncker, 1932; Costea et al., 2006a). The strong support for the monophyly of the section contrasts with the very short internal branch lengths (Stefanović et al., 2007) suggesting a recent and rapid diversification accompanied by the accumulation of repetitive DNA with rates that might be different even at population level. A similar case is *C. indecora* in section *Indecorae*, for which several varieties have been described in addition to other two species, *C. coryli* and *C. warneri* Yunck. (Yuncker, 1932; Costea et al., 2006b).

### The Increase of the Genome Size in Some Lineages of *Cuscuta* Is Probably Linked to Parasitism

Neumann et al. (2020) suggested that there is no correlation between genome size and the parasitic lifestyle of *Cuscuta*. However, according to the Plant DNA C-value Database, other species of Convolvulaceae have small genome sizes, with an average of approximately 1C = 0.97 Gbp and the largest value reported for the hexaploid *Ipomoea batatas* (L.) Lam. (2*n* = 90; 1C = 2.25 Gbp). Our estimation of the ancestral genome size of *Cuscuta* was ca. 1C = 12 Gbp, and 1C = 20 Gbp when including only *Cuscuta* species in the analyses. The phylogenetic position of subgenus *Monogynella* as sister to the rest of the genus, with such large genome sizes, may have resulted in an overestimation of the ancestral genome size for the genus as a whole. In spite of this possible bias, based on the limited Convolvulaceae data available, there was probably a genome expansion in subgenus *Monogynella*, and independent increases in other lineages, especially in sections *Indecorae* and *Oxycarpae* of subgenus *Grammica*.

The genome constraint hypothesis (Knight et al., 2005) suggests that the costs associated with the accumulation and replication of repetitive DNA reduce plant performance and negatively affects speciation and the distribution and abundance of species. Parasitic lifestyle eliminates the restrictions imposed by the growth rate of the meristem or the “genomic economy,” because they take resources from their hosts (Gruner et al., 2010; Piednoël et al., 2012). Thus, despite the genomic reductions associated with the loss of autotrophic functions (Revill et al., 2005; Banerjee and Stefanović, 2019), there is a tendency for parasitic plants to have larger and more complex genomes (reviewed by Lyko and Wicke, 2021). For example, in Orobanchaceae, the genomes of the autotrophic *Lindenbergia philippensis* (Cham. & Schltdl.) Benth. and the hemiparasite *Schwalbea americana* L. are much smaller than those of the holoparasite *Orobanche* L. and *Phelipanche* Pomel, which fits the hypothesis of larger genome sizes in parasitic plants (Gruner et al., 2010; Piednoël et al., 2012). Without the selective constraints imposed by the nutrient and energy economy (Gruner et al., 2010; Piednoël et al., 2012), genome size could vary *via* mechanisms such as mobile elements activation and ectopic recombination. The amplification and diversity of transposable elements can be influenced by the DNA transposition and elimination rates, population size, reproduction mode, host plant defense mechanism, and even horizontal gene transfer (Devos et al., 2002; Bourque et al., 2018; Biscotti et al., 2019; Nishihara, 2020). This variation may or may not be fixed by the action of genetic drift and natural selection and could possibly allow a wider range of variation including the upper limits of genome size in *Cuscuta*, which are usually selected against in green plants.

Unlike in Orobanchaceae, genome size increases in *Cuscuta* are, however, not strictly related to the evolution to holoparasitism within the genus. Subgenus *Monogynella* has the least reduced plastome and higher ability for carbon fixation than the rest of subgenera; however, it has the largest genomes. Different levels of plastome reduction have been documented for the genus (Banerjee and Stefanović, 2020), and fully holoparasitic species are known in section *Ceratophorae* (subgenus *Grammica*, clade K; Banerjee and Stefanović, 2019). Although no genome size estimations have been done for the section, the chromosomes are not significantly bigger than in other related sections except for an apparent trend toward asymmetrical karyotypes. Species of sect. *Subulatae* (clade O) are also holoparasitic (Braukmann et al., 2013) and the only known genome sizes for the section are those of *C. chilensis* Ker Gawl. and *C. purpurata* Phil., two diploids with intermediate genome size (1C = 2.80 and 2.96, respectively).

Smaller genomes theoretically facilitate faster cell divisions and therefore growth (Gruner et al., 2010) and cell division rates (Símova and Herben, 2012). In *Cuscuta*, however, there is no clear negative correlation between genome size and growth rate, possibly because it may also be influenced by other factors, such as cell elongation. *Cuscuta indecora*, with the largest known genome in subgenus *Grammica*, is an invasive weed and a seed contaminant of crop plants, with profuse and fast growth over its hosts (Cudney et al., 1992; Costea et al., 2006b). Although we have not performed specific comparative experiments, we did not observe any evident difference in growth rate between this species and, e.g., *C. australis*, the species with the smallest genome known in the genus, even though both were growing on the same host in the same greenhouse conditions. It is remarkable that *C. indecora* behaves as a fast-growing weed, despite theoretically having longer cell cycles in which the whole genome must be replicated. *Cuscuta indecora* and weedy species of section *Oxycarpae,* such as *C. gronovii*, might be model systems to study the correlation between higher metabolic activity necessary to maintain the growth rate and the accumulation of repetitive DNA and transposable elements contributing to genome upsizing.

### Changes in the Position of rDNA Sites May Indicate the Dynamics of Tandem Repetitive DNA Sequences in *Cuscuta*

Most *Cuscuta* species have a few rDNA sites: only one pair of 5S and one pair of 35S rDNA sites. Although the number of 5S and 35S rDNA loci is positively correlated with ploidy level (García et al., 2017), this does not hold true for this genus*. Cuscuta sandwichiana*, the highest polyploid reported, has one pair of 5S and two pairs of 35S rDNA sites, while phylogenetically close diploids, such as *C. australis*, have one pair of 5S and one pair of 35S rDNA sites. This may be due to the fact that some *Cuscuta* polyploids are interspecific hybrids, such as *C. sandwichiana* (Stefanović and Costea, 2008; García et al., 2014). The occurrence of recombination and gene conversion that results in the presence of rDNA copies from only one of the parents is often observed in hybrids. In allotetraploids of the *Dilatata* group of the genus *Paspalum* L. (Poaceae), for instance, the recovered ITS sequences show homogenization toward the paternal genome only (Vaio et al., 2019). In addition, the decrease in the number of expected sites may occur due to the elimination of some sites in terminal regions. The terminal position of the rDNA sites would be selectively favorable compared to the proximal ones, as it would reduce the chances of deleterious chromosomal rearrangements related to unequal recombination and recombination between non-homologous chromosomes (Roa and Guerra, 2012; García et al., 2017). Besides, the number of parental rDNA sites may be quite conserved in young, artificial allopolyploids. However, in natural allopolyploids, this number is often reduced, especially the 5S rDNA sites (Lee et al., 2011; Volkov, 2017). In *C. veatchii*, for example, there is a reduction in the rDNA sites in relation to its parents *C. denticulata* and *C. nevadensis* I.M. Johnst., indicating an old origin of this hybrid (Ibiapino et al., 2019).

It is common for the 5S and 35S rDNA sites to be found at separate locations in the genome, even on different chromosomes. This may be related to the fact that they are transcribed in different cellular compartments, by different enzymes (García and Kovařík, 2013). However, in *Cuscuta*, many species had rDNA sites located on the same chromosome, across all subgenera. In *C. monogyna* (subgenus *Monogynella*, sister to the rest of the genus), almost all its 30 chromosomes had 5S and 35S rDNA sites positioned closely, both on the same chromosome arm and on different arms. Co-occurrence of 5S and 35S rDNA sites on the same chromosome is higher in karyotypes with multiple sites and is frequently on the same arm (Roa and Guerra, 2015; García et al., 2017). However, all other *Cuscuta* species (e.g., *C. veatchii* and *C. indecora*) with sites on the same chromosome have these sites positioned on the same arm.

In plants, 5S rDNA usually occupies proximal and less frequently interstitial and terminal regions, while 35S rDNA tends to occupy terminal regions (Roa and Guerra, 2012, 2015). In *Cuscuta*, while 5S rDNA was more frequently found in interstitial regions, 35S is frequently found on peri/centromeric regions. In the only two holocentric species of *Cuscuta* whose rDNA sites are reported in this work, the positions of these sites also diverged from that found in other groups of plants. Generally, both 5S rDNA and 35S rDNA occupy terminal regions in holocentrics (Roa and Guerra, 2012, 2015). In holocentric *Cuscuta* species, only the 35S occupied a terminal position. The 5S rDNA sites were at more interstitial positions.

The ancestral character reconstruction suggested the interstitial position as ancestral for the 5S rDNA. This characteristic is present in all species, including *C. monogyna* (subgenus *Monogynella*) and *C. indecora* (subgenus *Grammica*), which also showed proximal and terminal sites, respectively. The 35S was more variable, and the “mix” condition, where rDNA sites were found in more than one location, was present at several clades throughout the phylogeny. The 35S rDNA is commonly pericentromeric; however, in species with more sites, additional sites are usually found in interstitial regions. In the subgenus *Cuscuta*, without a localized centromere, all 35S sites were terminal.

According to the Plant rDNA Database (García et al., 2017), within the Convolvulaceae, published rDNA site data are available only for seven species of *Ipomoea*, which is not closely related to *Cuscuta* and, thus, may not aid in resolving ancestral rDNA state. In the subgenus *Monogynella*, there is an increase in the diversity of positions in which the rDNA sites were found, suggesting that the “mix” condition is derived and, due to the amplification of these sites, the ribosomal DNAs began to occupy different positions along the chromosome. Recent studies show the possible influence of repetitive DNA amplification on genomic changes in the genus *Cuscuta* (Neumann et al., 2020; Ibiapino et al., 2022), indicating that the increase of these sites in *Monogynella* may be caused by the amplification of rDNA repeats as observed for other tandem repetitive sequences in the genus, and these are actually pseudogenes.

The tandem repetitive DNA in *Cuscuta* appears to be quite complex. For example, *C. europaea* have species-specific satellite DNA sequences such as the CUS-TR24, colocalized with centromeric proteins, also species-specific (Oliveira et al., 2020). Analysis using long reads showed a complex organization of CUS-TR24. The sequence of this satellite is interspersed with insertions mainly from LINE retrotransposons (Vondrak et al., 2021). In the subgenus *Pachystigma*, there is also evidence of these complex satellites. For example, the CnSat10-1,400 found in *C. nitida* in addition to being similar to a LINE type element, co-localizes with 35S signals in the chromosomes of this species. In addition, the most abundant SF1 family of *C. nitida* also co-locates with 35S signals (Ibiapino et al., 2022). This complex organization, with possible insertions of rDNA in other repetitive DNAs, could influence the diversity of number and position of rDNA sites in *Cuscuta*. In *Allium cepa* L., for example, it was shown that 35S rDNA is able to move from one locus to another in the genome. Furthermore, it is associated with telomeric DNA and other satellite DNAs, suggesting that the 35S of this species undergoes excision-reintegration mediated by these sequences (Mancia et al., 2015; Fu et al., 2019). Thus, the current data suggest that the evolution of rDNA in *Cuscuta* may be influenced by other tandem repeats or transposable elements.

## Conclusion

The data support the basic number *x* = 15 in *Cuscuta*, with duplications more common in subgenus *Grammica*. As expected, dysploidy occurred predominantly in the holocentric clade (subgenus *Cuscuta*). The remarkable increase and variation of the genome size in most lineages of *Cuscuta* may have been favored by the release of constraints enabled by its parasitic lifestyle. The data showed an expansion of genome size in comparison with the other Convolvulaceae, mostly by repetitive DNA amplification. This amplification of sequences may also have given rise to the great diversity of 5S and 35S ribosomal DNA sites found in the genus, and it seems to contribute to the emergence of “mix”-type karyotypes, in which multiple positions are occupied by these rDNA sites. This work analyzed data from 57 of the 200 *Cuscuta* species, which represents only 29% of *Cuscuta* species, indicating that the karyotypic diversity of the genus may be still greater than reported. Nevertheless, *Cuscuta* is one of the exceptionally diverse genera within the angiosperms in terms of karyotype and genome size. *Cuscuta*, having closely related species with different ploidy levels and marked differences in chromosome and genome size, is an excellent, tractable model system in which to study genome downsizing in polyploids as well as correlation of DNA content to phenotype such as pollen size, growth rate, cell cycle time, and epidermal cell size.

## Data Availability Statement

The datasets presented in this study can be found in online repositories. The names of the repository/repositories and accession number(s) can be found in the article/Supplementary Material.

## Author Contributions

AI performed the scientific experiments, data collection, and writing of the manuscript. MG contributed to the writing and reviewing of the manuscript and general discussions. BA performed all the phylogenetic analysis and supported the computational analysis. MB contributed by analyzing the data and reviewing the manuscript. MC and SS edited the manuscript, contributed to the collection, identification, and molecular data of the plant material, and discussions of the data. AP-H designed the experiments, supervised, and coordinated the project. All authors contributed to the article and approved the submitted version.

## Funding

We thank the Fundação de Amparo a Ciencia e Tecnologia de Pernambuco (FACEPE) for the financing of the Postgraduate scholarship; the Conselho Nacional de Desenvolvimento Científico e Tecnológico (CNPq); and the Coordenação de Aperfeiçoamento de Pessoal de Nível Superior (CAPES, Financial Code 001) for the financial support for the development of the project. BA thanks CAPES for the post-doc fellowship (process #88882.315044/2019-01). NSERC Discovery Canada supported the research of MC (327013) and SS (326439).

## Conflict of Interest

The authors declare that the research was conducted in the absence of any commercial or financial relationships that could be construed as a potential conflict of interest.

## Publisher’s Note

All claims expressed in this article are solely those of the authors and do not necessarily represent those of their affiliated organizations, or those of the publisher, the editors and the reviewers. Any product that may be evaluated in this article, or claim that may be made by its manufacturer, is not guaranteed or endorsed by the publisher.

## Footnotes

^1^ https://pastml.pasteur.fr/

## Abbreviations

AIC: Akaike Information Criterion
CMA: Chromomycin A3
DAPI: 4,6-Diamidino-2-phenylindole
FISH: Fluorescence *in situ* hybridization
PCR: Polymerase chain reaction
rDNA: Ribosomal DNA
BBM: Bayesian Binary MCMC
RASP: Reconstruct Ancestral State in Phylogenies
