# Supplementary material for: The Evolution of Cytogenetic Traits in Cuscuta (Convolvulaceae), the Genus With the Most Diverse Chromosomes in Angiosperms
Source: Front Plant Sci. 2022 Apr 1;13:842260. doi: 10.3389/fpls.2022.842260 (PMC9011109; doi:10.3389/fpls.2022.842260)
Supplement: Supplementary file 1 [file Table_1.pdf]

Supplementary Table 1: Access data of the sequences used to perform phylogenetic reconstruction (infrageneric classification from Costea et al. 2015a).

Subgenus	Section	Species	rbcL	26S	trnL-F	nrITS
Cuscuta	*Cuscuta*	*Cuscuta approximata*	KJ436605	KJ400040	EF202557	KY020426
Cuscuta	*Babylonicae*	*Cuscuta babylonica*	KJ436611	KJ400047		KU761258
Cuscuta	*Epistigma*	*Cuscuta capitata*	KJ436615	KJ400051		DQ924584
Cuscuta	*Cuscuta*	*Cuscuta epilinum*	KJ436641	KJ400079	AY558849	DQ924610
Cuscuta	*Cuscuta*	*Cuscuta epithymum*	KJ436643	KJ400080	KC569804	
Cuscuta	*Cuscuta*	*Cuscuta europaea*	AY101060	KJ400082	AY558851	AY554401
Cuscuta	*Epistigma*	*Cuscuta pedicellata*	KJ436700	KJ400151		DQ924582
Cuscuta	*Cuscuta*	*Cuscuta planiflora*	KJ436702	KJ400153	AY558858	AY558822
Grammica	*Obtusilobae*	*Cuscuta americana*	KJ436602	KJ400038	EF194398	EF194597
Grammica	*Cleistogrammica*	*Cuscuta australis*	KJ436607	KJ400043	EF194457	EF194667
Grammica	*Ceratophorae*	*Cuscuta bonafortunae*		JN234780	JN234799	JN234809
Grammica	*Californicae*	*Cuscuta brachycalyx*	EU883441	EU883489		
Grammica	*Californicae*	*Cuscuta californica*	EU883445	EU883493	EF194479	EF194691
Grammica	*Cleistogrammica*	*Cuscuta campestris*	EU883476	KJ400050	EF194453	EF194661
Grammica	*Oxycarpae*	*Cuscuta cephalanthi*	KJ436618	KJ400054	EF194413	EF194632
Grammica	*Ceratophorae*	*Cuscuta chapalana*	KJ436620	JN234783	EF194338	EF194578
Grammica	*Subulatae*	*Cuscuta chilensis*		KJ400056		EF194523
Grammica	*Grammica*	*Cuscuta chinensis*	KJ436621	KJ400059	EF194368	
Grammica	*Oxycarpae*	*Cuscuta compacta*	KJ436626	KJ400064	EF194426	EF194640
Grammica	*Indecorae*	*Cuscuta coryli*	KJ436629	KJ400067	EF194288	EF194539
Grammica	*Prismaticae*	*Cuscuta corymbosa* var. *grandiflora*	KJ436630	KJ400068	EF194343	EF194584
Grammica	*Ceratophorae*	*Cuscuta costaricensis*	KJ436633	JN234786	EF194340	EF194580
Grammica	*Lobostigmae*	*Cuscuta cotijana*	KJ436635	KJ400072	KC485355	KC485379
Grammica	*Oxycarpae*	*Cuscuta cuspidata*	KJ436637	KJ400075	EF194429	EF194643

Grammica	*Denticulatae*	*Cuscuta denticulata*	KJ436639	KJ400077	EF194411	EF194627
Grammica	*Umbellatae*	*Cuscuta desmouliniana*	KJ436640	KJ400078	EU288341	EU288359
Grammica	*Ceratophorae*	*Cuscuta erosa*		JN234789	JN234804	EF194574
Monogynella	*Monogynella*	*Cuscuta exaltata*	KJ436645	KJ400084		EU330323
Grammica	*Gracillimae*	*Cuscuta globosa*			OL362010	OL362011
Grammica	*Oxycarpae*	*Cuscuta glomerata*	KJ436651	KJ400094	EF194431	EF194644
Grammica	*Subulatae*	*Cuscuta grandiflora*		KJ400097		EF194535
Grammica	*Oxycarpae*	*Cuscuta gronovii*	KJ436654	EU883530	EF194422	EF194639
Grammica	*Californicae*	*Cuscuta howelliana*	EU883456	EU883505	EF194504	EF194717
Grammica	*Indecorae*	*Cuscuta indecora*	KJ436664	KJ400107	EF194300	EF194549
Grammica	*Indecorae*	*Cuscuta indecora* var. *neuropetala*			EF194301	EF194544
Grammica	*Denticulatae*	*Cuscuta nevadensis*	KJ436689	KJ400136	EF194408	EF194630
Grammica	*Cleistogrammica*	*Cuscuta obtusiflora*	KJ436693	KJ400140	EF194463	EF194673
Grammica	*Californicae*	*Cuscuta occidentalis*	EU883459	EU883509	EF194477	EF194695
Grammica	*Californicae*	*Cuscuta pacifica*	EU883463	EU883513		
Grammica	*Partitae*	*Cuscuta partita*	KJ436698	KJ400149	EF194353	EF194591
Grammica	*Cleistogrammica*	*Cuscuta pentagona*	KJ436701	KJ400152	EF194467	EF194679
Grammica	*Cleistogrammica*	*Cuscuta polygonorum*			KT371735	KT383292
Grammica	*Denticulatae*	*Cuscuta psorothamnensis*			MH920299	MH923173
Grammica	*Subulatae*	*Cuscuta purpurata*		KJ400159		EF194526
Grammica	*Racemosae*	*Cuscuta racemosa*			EF194449	
Grammica	*Oxycarpae*	*Cuscuta rostrata*	KJ436709	KJ400162	EF194428	EF194642
Grammica	*Californicae*	*Cuscuta salina*	EU883465	EU883515	GQ254882	GQ254890
Grammica	*Cleistogrammica*	*Cuscuta sandwichiana*	KJ436712	KJ400165	EU288333	EU288356
Grammica	*Gracillimae*	*Cuscuta sidarum*	KJ436713	KJ400167	EF194309	EF194553
Grammica	*Californicae*	*Cuscuta subinclusa*	EU883470	EU883521	EF194491	EF194703
Grammica	*Lobostigmae*	*Cuscuta tinctoria*	KJ436721	KJ400175	EF194394	EF194618
Grammica	*Lobostigmae*	*Cuscuta tinctoria* var. *floribunda*	KJ436723	KJ400177		

Grammica	*Oxycarpae*	*Cuscuta umbrosa*	KJ436728	KJ400182	EF194435	EF194646
Grammica	*Denticulatae*	*Cuscuta veatchii*	KJ436730	KJ400184	MH920306	MH923183
Grammica	*Lobostigmae*	*Cuscuta volcanica*	KJ436734	KJ400188	KC485376	KC485421
Monogynella	*Monogynella*	*Cuscuta japonica*	AY101061	KJ400114	AY101170	KP015829
Monogynella	*Monogynella*	*Cuscuta lupuliformis*	KJ436680	KJ400126	AY558854	KU707914
Monogynella	*Monogynella*	*Cuscuta monogyna*	KJ436686	KJ400133	MH115452	MH109269
Monogynella	*Monogynella*	*Cuscuta reflexa*	KJ436708		AY558859	AY558823
Pachystigma	*Pachystigma*	*Cuscuta africana*	KJ436602	KJ400037		DQ924574
Pachystigma	*Pachystigma*	*Cuscuta angulata*	KJ436602	KJ400039	EF152065	DQ924575
Pachystigma	*Pachystigma*	*Cuscuta nitida*	KJ436690	KJ400137	EF202558	EF202562
OUTGROUPS						
Convolvulaceae		*Calystegia hederacea Wall.*	LC085875			LC225748
Convolvulaceae		*Calystegia sepium* (L.) R. Br.	MH657526	AF148267	AY101101	MN601829
Convolvulaceae		*Convolvulus althaeoides L.*	KC529175		KC786129	KC528819
Convolvulaceae		*Convolvulus arvensis L.*	MH536594	AF479176	AY101102	AY560274
Convolvulaceae		*Convolvulus canariensis* L.	MN783776			AY560276
Convolvulaceae		*Convolvulus cantabricus L.*	MT828358			KC528967
Convolvulaceae		*Convolvulus farinosus L.*	KC529210			KC528841
Convolvulaceae		*Convolvulus floridus L.*	KC529214		MF621882	EF371754
Convolvulaceae		*Convolvulus glomeratus Choisy*	KC529219		KC786132	KC528887
Convolvulaceae		*Convolvulus lineatus L.*	KC529239			KC528963
Convolvulaceae		*Convolvulus perraudieri Coss.*	MN783780			
Convolvulaceae		*Convolvulus scoparius* L.	MT828356			
Convolvulaceae		*Cressa cretica L.*	KY860700			
Convolvulaceae		*Dichondra repens J. R. Forst. & G. Forst.*	MN379753		HQ412975	KJ004289
Convolvulaceae		*Dinetus racemosus (Roxb.) B. -Ham. ex Sweet*	HQ384920			MN380241
Convolvulaceae		*Evolvulus alsinoides (L.) L.*	MH767511	AF148499	AY101146	MG730315
Convolvulaceae		*Jacquemontia tamnifolia (L.) Griseb.*	AY101037	KJ400199	AY206764	MH768118

Montiniaceae	*Montinia caryophyllacea Thunb.*	L11194		AY101142	DQ219865
Convolvulaceae	*Neuropeltis acuminata (P. Beauv.) Benth.*	AY101033		AY101160	
Solanaceae	*Porana paniculata Roxb.*	AY101051	KJ400202	AY101172	
Solanaceae	*Schizanthus pinnatus Ruiz & Pav.*	U08619			DQ299453
