# Supplementary material for: The Evolution of Cytogenetic Traits in Cuscuta (Convolvulaceae), the Genus With the Most Diverse Chromosomes in Angiosperms
Source: Front Plant Sci. 2022 Apr 1;13:842260. doi: 10.3389/fpls.2022.842260 (PMC9011109; doi:10.3389/fpls.2022.842260)
Supplement: Supplementary file 2 [file Data_Sheet_1.PDF]

Supplementary figures

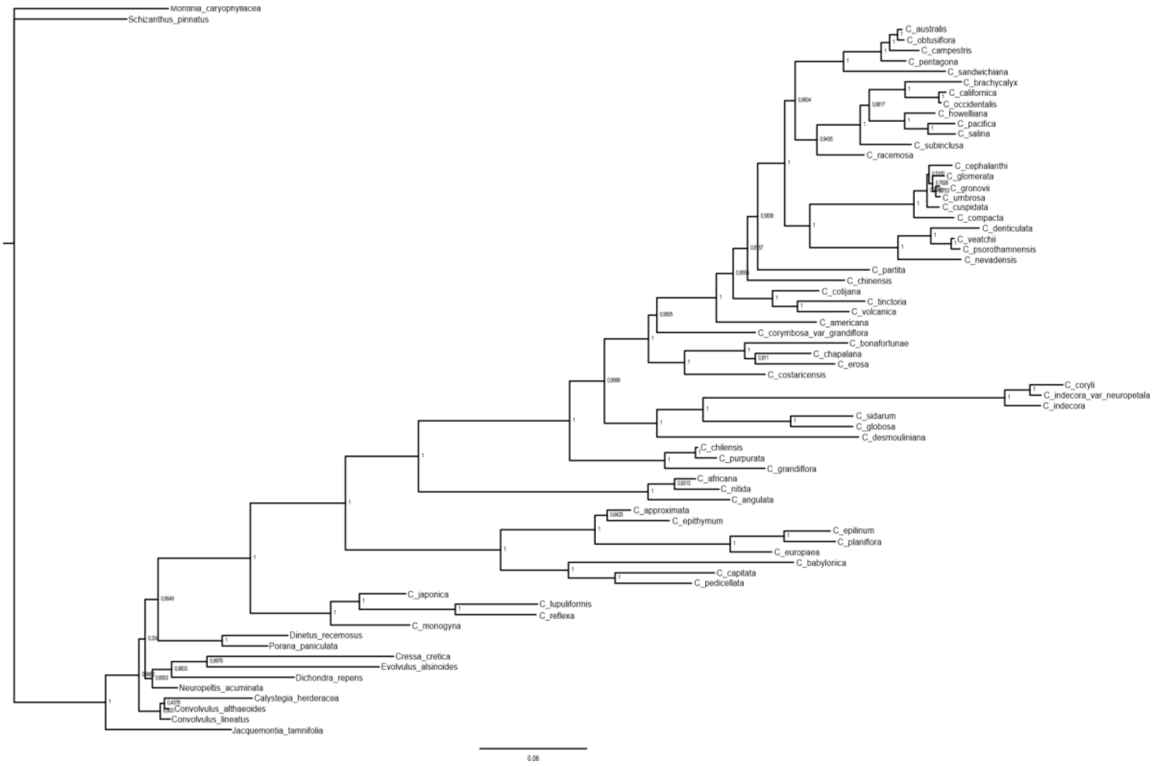

Supplementary figure 1: Consensus tree derived from the combination of *rbc*L, *trn*L-*trn*F, nrITS and 26S sequences made by Bayesian inference. The genus *Cuscuta* is monophyletic and represented by 58 taxa and 57 species.

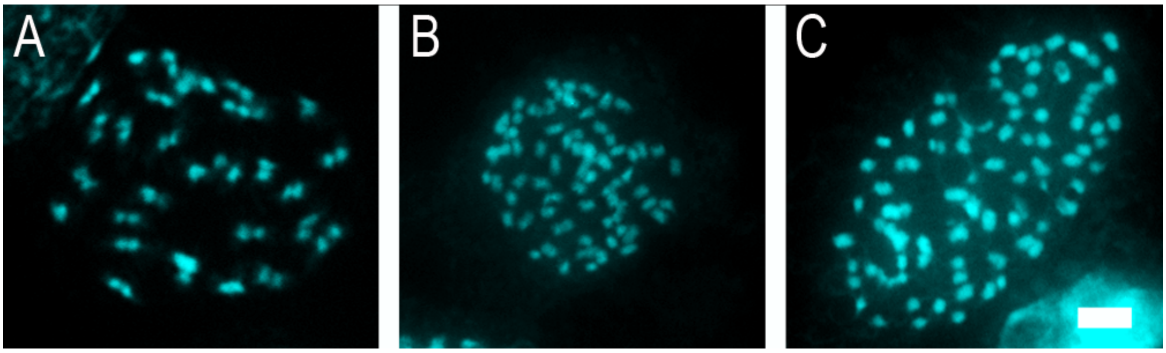

Supplementary figure 2: Mitotic metaphases corresponding to new chromosome counts reported in this work. **A**) *Cuscuta partita* (2*n* = 30); **B**) a polyploid cytotype of *C. cotijana* 2*n* = 60; and **C**) *C. globosa* with 2*n* = 90. Bar in **C** represents 5µm

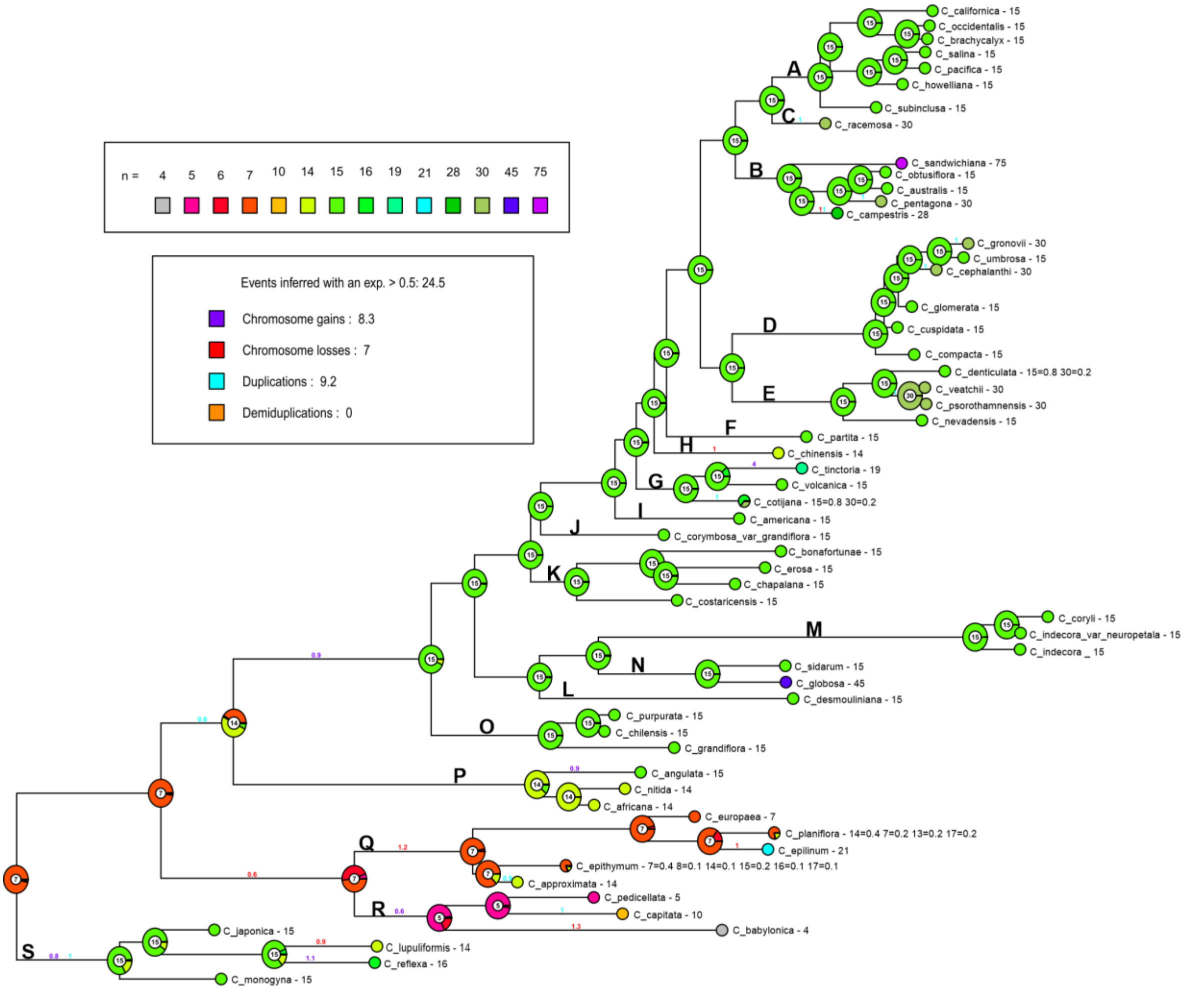

Supplementary figure 3: Reconstruction of the chromosome number evolution in *Cuscuta* with the BASE_NUM_DUPL model. The pie charts on the nodes represent the probability of each inferred chromosome number, the numbers along the branches represent the probability of frequencies of the inferred events (gains, losses, duplications and demiduplications) The bold letters represent the clades described by García et al. (2014): **S** (subgenus *Monogynella*), **R** and **Q** (subgenus *Cuscuta*), **P** (subgenus *Pachystigma*) and **A - O** (subgenus *Grammica*)

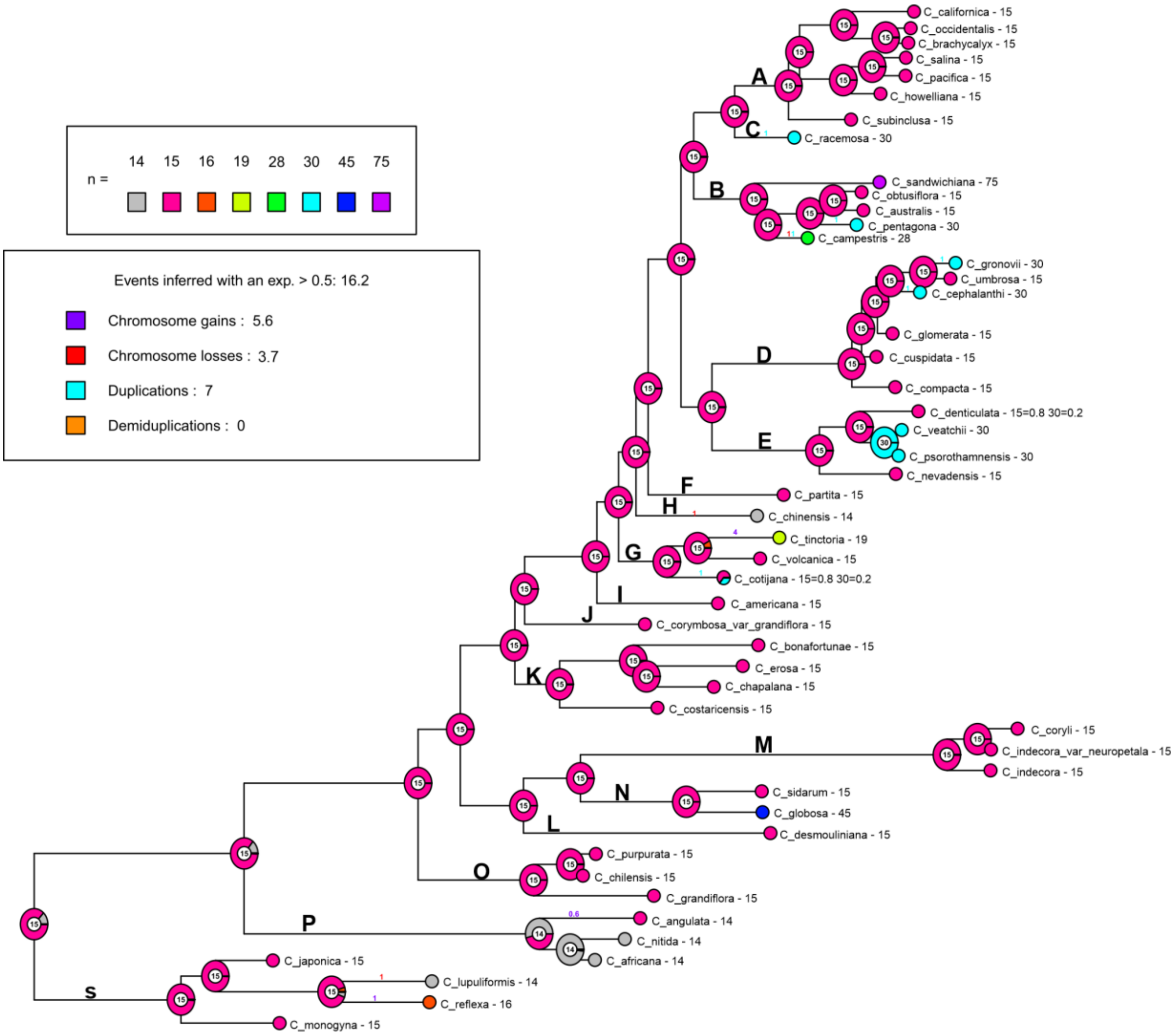

Supplementary figure 4: Reconstruction of the chromosome number evolution in *Cuscuta* with the BASE_NUM_DUPL model, without subgenus *Cuscuta*. The pie charts on the nodes represent the probability of each inferred chromosome number, the numbers along the branches represent the probability of frequencies of the inferred events (gains, losses, duplications and de-duplications). The bold letters represent the clades described by García et al. (2014): **S** (subgenus *Monogynella*), **R** and **Q** (subgenus *Cuscuta*), **P** (subgenus *Pachystigma*) and **A** - **O** (subgenus *Grammica*)

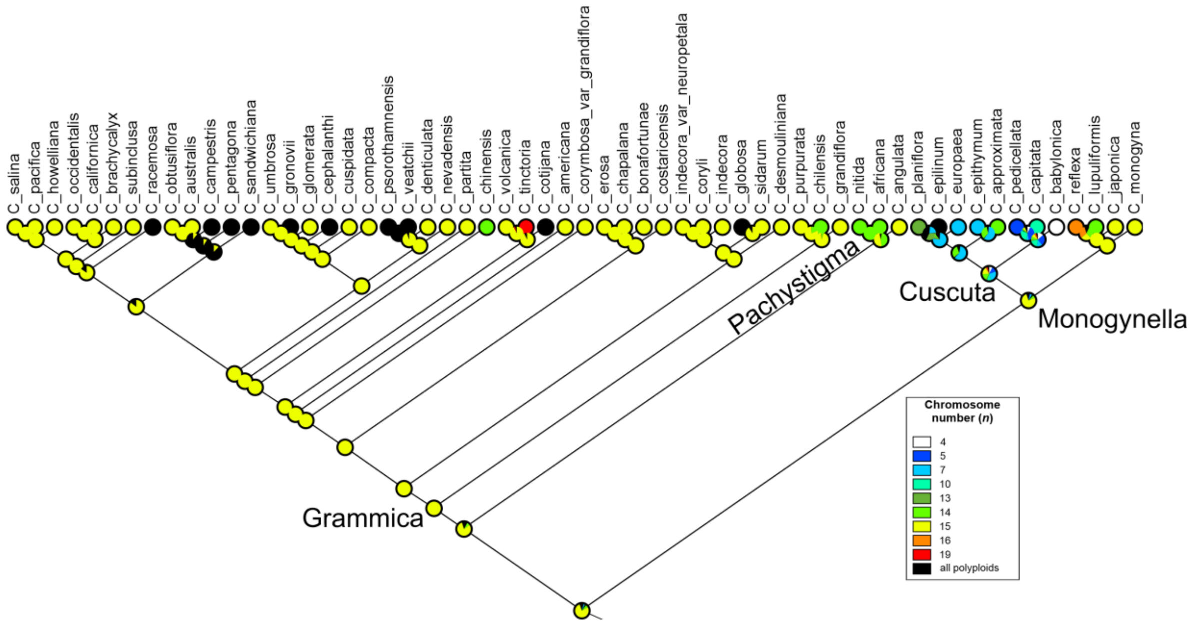

Supplementary figure 5: Reconstruction of ancestral chromosome number in *Cuscuta* performed by Mesquite. The reconstruction method used was of maximum likelihood. Each subgenus is indicated in the tree.

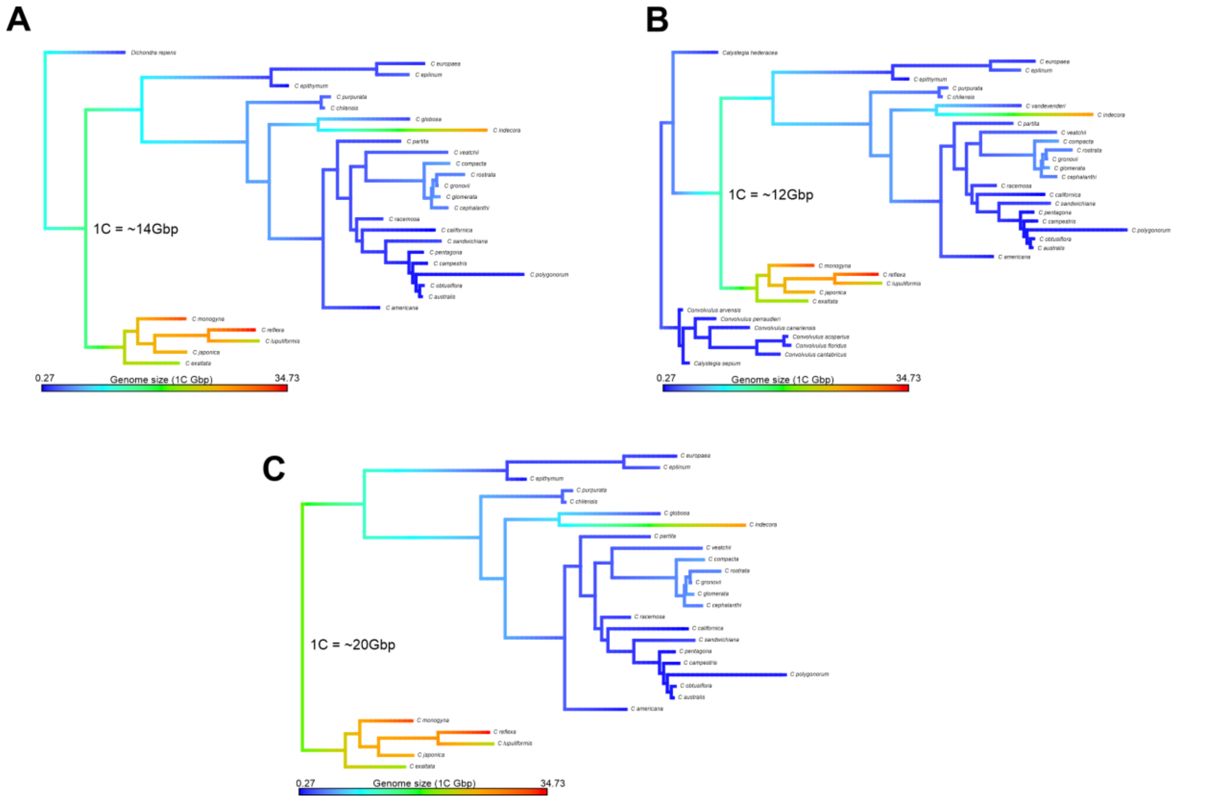

Supplementary figure 6: Reconstructions of the genome size evolution generated by Phytools in R. The variation is shown in a colour scale: the largest genomes in shades of red and the smallest genomes in shades of blue. In A, a species of the genus *Dichondra* was added as an outgroup. In B, two species of *Calystegia* and six species of *Convolvulus* were used as the outgroup. In both examples the ancestral genome size in *Cuscuta* was smaller than in the analysis considering only *Cuscuta*, without outgroups (C). Despite this, it is shown that, in relation to the other Convolvulaceae, there was an expansion of the genome in *Cuscuta*

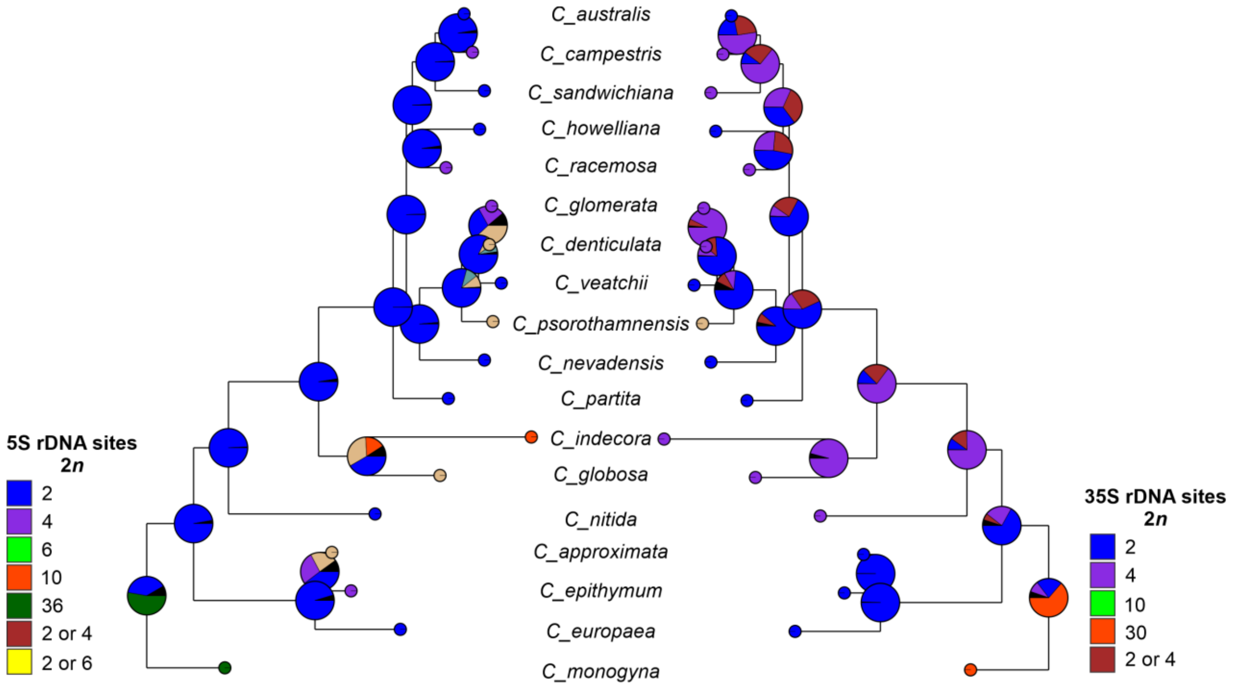

Supplementary figure 7: Ancestral 5S and 35S rDNA site number reconstruction performed in RASP. The pie charts represent the probability of each state (number of site) in each of the nodes of the phylogeny. The results show numerous sites in both 5S (36 sites) and 35S (30 sites) showing the influence of *C. monogyna* (subgenus *Monogynella*), the species with the highest number of rDNA sites, in the analysis.
